# Tumors driven by *RAS* signaling harbor a natural vulnerability to oncolytic virus M1

**DOI:** 10.1002/1878-0261.12820

**Published:** 2020-10-25

**Authors:** Jing Cai, Kaiying Lin, Wei Cai, Yuan Lin, Xincheng Liu, Li Guo, Jifu Zhang, Wencang Xu, Ziqing Lin, Chun Wa Wong, Max Sander, Jun Hu, Guangmei Yan, Wenbo Zhu, Jiankai Liang

**Affiliations:** ^1^ Department of Pharmacology Zhongshan School of Medicine Sun Yat‐sen University Guangzhou China; ^2^ Guangzhou Virotech Pharmaceutical Co., Ltd. Guangzhou China

**Keywords:** biomarker, cancer accurate therapy, CDKN1A, M1, oncolytic virus, RAS

## Abstract

Oncolytic viruses are potent anticancer agents that replicate within and kill cancer cells rather than normal cells, and their selectivity is largely determined by oncogenic mutations. M1, a novel oncolytic virus strain, has been shown to target cancer cells, but the relationship between its cancer selectivity and oncogenic signaling pathways is poorly understood. Here, we report that *RAS* mutation promotes the replication and oncolytic effect of M1 in cancer, and we further provide evidence that the inhibition of the RAS/RAF/MEK signaling axis suppresses M1 infection and the subsequent cytopathic effects. Transcriptome analysis revealed that the inhibition of RAS signaling upregulates the type I interferon antiviral response, and further RNA interference screen identified CDKN1A as a key downstream factor that inhibits viral infection. Gain‐ and loss‐of‐function experiments confirmed that CDKN1A inhibited the replication and oncolytic effect of M1 virus. Subsequent TCGA data mining and tissue microarray (TMA) analysis revealed that CDKN1A is commonly deficient in human cancers, suggesting extensive clinical application prospects for M1. Our report indicates that virotherapy is feasible for treating undruggable *RAS*‐driven cancers and provides reliable biomarkers for personalized cancer therapy.

## Graphical abstract

RAS/RAF/MAPK/p‐ERK signaling promotes the replication and oncolytic effect of M1 virus via inhibiting P21 and subsequent suppression of Cyclin‐dependent kinase 2 (CDK2). Inhibition of RAS/RAF/MAPK/p‐ERK signaling by sorafenib or U0126 suppresses the oncolytic effect of M1. Knockout of p21 enhances the effect of M1, while overexpression of p21 inhibits it. Inhibition of CDK2 by K03861 attenuates the efficacy of M1.

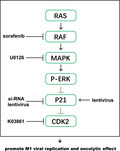

**Abbreviations**
CCLE: Cancer Cell Line Encyclopedia
CDK2: Cyclin‐dependent kinase 2
Cl‐casp3: Cleaved caspase‐3
FDA: Food and Drug Administration
GSEA: Gene set enrichment analysis
IFN: Interferon
IHC: Immunohistochemistry
IRGs: IFN‐regulated genes
MOI: Multiplicity of infection
NC: Negative control
PFU: Plaque‐forming unit
RPPA: Reversed‐phase protein array
TMA: Tissue microarray

## 1 Introduction

Oncolytic viruses offer a new approach for cancer therapy, which exploits tumor mutations to specifically replicate within and kill tumor cells without causing harm to normal cells [1, 2, 3, 4, 5]. Oncolytic viruses also activate the antitumor immune response in addition to direct killing [6, 7, 8]. Since Talimogene laherparepvec became the first oncolytic virus approved by the US Food and Drug Administration (FDA) [9], numerous clinical trials have begun [10] and are expected to further boost the development of oncolytic therapies.

The tumor selectivity of oncolytic viruses is largely conferred by tumor‐specific aberrations in signaling pathways that normally sense and block viral replication. It is now well established that cancer‐specific aberrations in *BCL‐2*, *WNT*, *EGFR*, *RAS*, *TP53*, *RB1*, *PTEN,* and other cancer‐related genes predispose cancer cells to viral infection [7, 8, 11]. For example, Newcastle disease virus targets cancer cells overexpressing *BCL‐XL,* which prevents apoptosis and thereby permits the virus to utilize the transcription and translation machinery for the synthesis of the viral nucleocapsid [12]. The activation of *WNT* signaling, a key pathway in embryonic development that directs cell proliferation, polarity, and developmental fate, has been found to attenuate the host antiviral response and facilitate the infection and replication of several kinds of viruses [13, 14, 15]. In addition, cancer cells with *RAS* mutations cannot activate the PKR pathway which functions to prevent the production and spread of virus, rendering cancer cells permissive to reovirus, herpesvirus, and vaccinia virus infection [16, 17, 18, 19].

M1 virus is an enveloped alphavirus with an 11.7 kb positive single‐stranded RNA genome [20], which contains four nonstructural proteins and five structural proteins. Our previous studies demonstrated that M1 is a potent oncolytic virus that selectively targets and induces irreversible endoplasmic reticulum stress‐mediated apoptosis in different cancers *in vitro* and *in vivo* [21, 22, 23]. More interestingly, the oncolytic effect of M1 can be enhanced by small‐molecule compounds, including BCL‐XL inhibitors, Smac mimetics, and DNA‐PK inhibitors [24, 25, 26, 27, 28, 29]. Our previous study established that M1 virus is a promising oncolytic virus for clinical cancer therapy. Though we have identified that the deficiency of zinc‐finger antiviral protein mediates the cancer selectivity of M1, however, the relationship between the cancer selectivity of M1 virus and oncogenic signals has not yet been illuminated.

In this study, we analyzed the relationship between viral infection and oncogenic mutations in 52 tumor cell lines and found that among the mutations in these cell lines, *K‐RAS* aberration promotes viral infection. Further expression profiling identified CDKN1A as a key factor downstream of the RAS/RAF/MEK signaling pathway that inhibits the replication of M1 virus. The knockdown of CDKN1A enhances the oncolytic effect of M1 virus in nude mice bearing human tumor cells, which largely represents the characteristics of human cancer. This study identifies *RAS* mutation and deficiency of CDKN1A as candidate biomarkers for personalized anticancer virotherapy.

## 2 Materials and methods

### 2.1 Cell culture and M1 viruses

All cell lines were purchased from the American Type Culture Collection (ATCC, Manassas, VA, USA). Cells were cultured in Dulbecco's Modified Eagle's medium supplemented with 10% (vol/vol) FBS (Thermo Fisher Scientific, Waltham, MA, USA) and 1% penicillin/streptomycin (Thermo Fisher Scientific). All cell lines were cultured at 37 °C in a 5% CO_2_ environment. All cell lines were authenticated by the short tandem repeat assay and were mycoplasma free according to the MycoGuard Mycoplasma PCR Detection Kit (MPD‐T‐050; GeneCopoeia, Rockville, MD, USA).

The M1 virus was grown in the Vero cell line and collected for experiments. The M1‐c6v1 strain of virus was provided by Guangzhou Virotech Pharmaceutical Co., Ltd, Guangzhou, Guangdong, China. M1‐GFP is a recombinant M1 engineered to express jellyfish green fluorescent protein [28]. The viral titer was determined by the TCID50 method using the BHK‐21 cell line and converted to plaque‐forming unit (PFU).

### 2.2 Lentiviruses and infections

Lentiviruses containing the CDKN1A (Gene ID: 1026) ORF (LPP‐G0313‐Lv242‐100) and shRNA (pLKD‐CMV‐mcherry‐2A‐Puro‐U6‐CDKN1A shRNA) of CDKN1A were constructed and packaged by GeneCopoeia and OBiO Technology, Shanghai, China. The HCT‐15 cell line was transfected with lentiviruses containing 5 μg·mL^−1^ polybrene (Sigma‐Aldrich, St. Louis, MO, USA); the multiplicity of infection (MOI) was 1. Three days after viral transfection, cells were selected with 1 μg·mL^−1^ puromycin for 7–14 days to establish a CDKN1A stably expressing cell line.

### 2.3 Cell viability assay

Cells were seeded in 96‐well plates at 3000 cells per well. After different treatments indicated in the figure legends were administered, 3‐(4,5‐dimethylthiazol‐2‐yl)‐2,5‐diphenyltetrazolium bromide (MTT) was added (1 mg·mL^−1^) and incubated at 37 °C for 3 h. The supernatants were removed, and the MTT precipitate was dissolved in 100 μL of DMSO. The optical absorbance was determined at 570 nm by a microplate reader (Synergy H1; Gene Company, Hong Kong, China).

### 2.4 Antibodies and reagents

The following antibodies and reagents were used in this study: ERK (#4695; Cell Signaling Technology, Danvers, MA, USA, RRID: AB_390779); p‐ERK (#4370; Cell Signaling Technology, RRID: AB_2315112); CDKN1A (#2947; Cell Signaling Technology, RRID: AB_823586); Ki‐67 (#9449; Cell Signaling Technology, RRID: AB_2797703); cleaved caspase‐3 (Cl‐casp3) (#9664; Cell Signaling Technology, RRID: AB_2070042); E1 and NS3 (Beijing Protein Innovation, Beijing, China); sorafenib (#S7397; Selleckchem, Houston, TX, USA); U0126 (#S1102; Selleckchem); cobimetinib (#S8041; Selleckchem); trametinib (S2673; Selleckchem); K03861 (#S8100; Selleckchem); polybrene (Sigma‐Aldrich); and puromycin (Thermo Fisher Scientific).

### 2.5 Expression profiling

HCT 15 tumor cells were treated with control, M1 (MOI = 1 pfu/cell), U0126 (16 μm), or M1 (MOI = 1 pfu/cell) plus U0126 (16 μm) for 24 h. Total RNA was extracted from 1 × 10^6^ cells with TRIzol reagent (Thermo Fisher Scientific) and was sent to CapitalBio (Beijing, China) for labeling and hybridization on the Affymetrix GeneChip Human Genome U133 Plus 2.0 Array.

### 2.6 RNA interference

siRNAs specific to different genes and control nontargeting siRNA were synthesized by Sigma‐Aldrich. The cells were transfected with the siRNAs (50 nm) using Lipofectamine RNAiMAX (Thermo Fisher Scientific) in Opti‐MEM medium (Thermo Fisher Scientific). The sequences of the siRNAs are listed below.

si‐CDKN1A 001: 5′‐TGATCTTCTCCAAGAGGAA‐3′

si‐CDKN1A 002: 5′‐GAATGAGAGGTTCCTAAGA‐3′

si‐CDKN1A 003: 5′‐TGGCGGGCTGCATCCAGGA‐3′

si‐IFIT3 001: 5′‐GACGGAATGTTATCAGACA‐3′

si‐IFIT3 002: 5′‐GGATAATCACCCAGAGAAA‐3′

si‐IFIT3 003: 5′‐CCAGAGAGCTCCTCTCTAA‐3′

si‐IFI27 001: 5′‐CTCTCCGGATTGACCAAGT‐3′

si‐IFI27 002: 5′‐CTGTCATTGCGAGGTTCTA‐3′

si‐IFI27 003: 5′‐CCAGGATTGCTACAGTTGT‐3′

si‐MX2 001: 5′‐GCACGATTGAAGACATAAA‐3′

si‐MX2 002: 5′‐GGGACGCCTTCACAGAATA‐3′

si‐MX2 003: 5′‐GGAGAATGAGACCCGTTTA‐3′

si‐ID1 001: 5′‐GAACTCGGAATCCGAAGTT‐3′

si‐ID1 002: 5′‐CACGTCATCGACTACATCA‐3′

si‐ID1 003: 5′‐TCAGGGACCTTCAGTTGGA‐3′

### 2.7 RT‐qPCR

Total RNA was extracted using TRIzol (Thermo Fisher Scientific), and 2 μg of total RNA was reverse‐transcribed to cDNA with oligo (dT) (synthesized by Thermo Fisher Scientific) and RevertAid Reverse Transcriptase (Thermo Fisher Scientific). The expression levels of the specific genes were calculated by the comparative C*_t_* method using SuperReal PreMix SYBR Green (FP204‐02; TIANGEN, Beijing, China) and an Applied Biosystems 7500 Fast Real‐Time PCR System (Thermo Fisher Scientific, RRID: SCR_014596). The sequences of the primers are listed below.

ID1 Forward: 5′‐CTGCTCTACGACATGAACGG‐3′

ID1 Reverse: 5′‐GAAGGTCCCTGATGTAGTCGAT‐3′

DDIT4 Forward: 5′‐TGAGGATGAACACTTGTGTGC‐3′

DDIT4 Reverse: 5′‐CCAACTGGCTAGGCATCAGC‐3′

CYP1B1 Forward: 5′‐AAGTTCTTGAGGCACTGCGAA‐3′

CYP1B1 Reverse: 5′‐GGCCGGTACGTTCTCCAAAT‐3′

IFI27 Forward: 5′‐TGCTCTCACCTCATCAGCAGT‐3′

IFI27 Reverse: 5′‐CACAACTCCTCCAATCACAACT‐3′

CDKN1A Forward: 5′‐CGATGGAACTTCGACTTTGTCA‐3′

CDKN1A Reverse: 5′‐GCACAAGGGTACAAGACAGTG‐3′

MX2 Forward: 5′‐CAGAGGCAGCGGAATCGTAA‐3′

MX2 Reverse: 5’‐TGAAGCTCTAGCTCGGTGTTC‐3′

ATP10D Forward: 5′‐GTGGTGGTCCTTACAATTATCGC‐3′

ATP10D Reverse: 5′‐CCCAACAGTAACGTCTTTCCAG‐3′

PNRC1 Forward: 5′‐ACTTGCCACTAACCAAGATCAC‐3′

PNRC1 Reverse: 5′‐TTGGAAGAACACTAGGAGAAGGT‐3′

JUN Forward: 5′‐AACAGGTGGCACAGCTTAAAC‐3′

JUN Reverse: 5′‐CAACTGCTGCGTTAGCATGAG‐3′

### 2.8 Western blot analysis

Cell samples were prepared using M‐PER Mammalian Protein Extraction Reagent (Thermo Fisher Scientific) and then separated by SDS/PAGE. The membranes were visualized with a ChemiDoc XRS + System (Bio‐Rad, Hercules, CA, USA) using Immobilon Western Chemiluminescent HRP Substrate (Millipore, Darmstadt, Germany).

### 2.9 Animal models

The mouse study was approved by the Animal Ethics and Welfare Committee of Sun Yat‐sen University, and all experiments were conducted according to the US ‘Public Health Service Policy on Humane Care and Use of Laboratory Animals’. HCT‐15‐negative control (NC) and HCT‐15‐shCDKN1A (5 × 10^6^ cells/mouse) tumor cells were implanted subcutaneously into the hind flanks of 5‐week‐old 16 g female BALB/c‐nu/nu mice (the mice were bought from Nanjing Biomedical Research Institute, China, and housed in an SPF facility with normal temperature and food). After 6 days, tumors were observed (~ 50 mm^3^). M1 virus (3.48 × 10^8^ TCID50 per mouse) was injected intravenously for 14 days. The lengths and widths of the tumors were measured every 3 days, and the tumor volume was calculated according to the formula (length × width^2^)/2. At the termination of the experiment, all mice were euthanized by overdose anesthesia, and the tumors were removed and fixed in 4% paraformaldehyde for subsequent immunohistochemistry (IHC) assays. The study was randomized and blind.

For detection of M1 viral copy number in tumor tissues, 3 days after the first medication, three mice in M1 virus‐treated HCT‐15‐NC and HCT‐15‐shCDKN1A tumor groups were euthanized by overdose anesthesia, and tumors were stripped out. Total RNA in tumors was extracted by Eastep® Super Total RNA Extraction Kit (Promega, Madison, Wisconsin, USA). Viral copy numbers were detected by TaqMan qRT‐PCR with FastKing One Step RT‐qPCR Kit (TIANGEN) according to the manufacturer's instructions. The sequence of primers and probe was listed as below:

Forward primer: 5′‐GGGATTCACTACACCTGCTTAGAC‐3′

Reverse primer: 5′‐GCTGACTCTGTCTGCGTAACC‐3′

Prober: 5′‐CTCTCATCAGCAGCGAGCCTCCT‐3′

### 2.10 Immunohistochemistry (IHC) assay

The expression of Ki‐67 and cleaved caspase 3 in the tumors was assessed by IHC. Briefly, tumor sections were dewaxed in xylene, hydrated in decreasing concentrations of ethanol, immersed in 0.3% H_2_O_2_‐methanol for 30 min, washed with phosphate‐buffered saline, and probed with monoclonal antibodies or isotype controls at 4 °C overnight. After being washed, the sections were incubated with biotinylated goat anti‐rabbit or anti‐mouse IgG at room temperature for 2 h. Immunostaining was visualized with streptavidin/peroxidase complex and diaminobenzidine, and sections were counterstained with hematoxylin.

### 2.11 Tissue microarray (TMA)

TMAs were purchased from Shanghai Biochip, Shanghai, China. IHC staining was performed on 5‐μm sections of the TMAs with CDKN1A antibody (#2947; Cell Signaling Technology, RRID: AB_823586). TMA slides were scanned using an Aperio slide scanner, and the staining intensity of CDKN1A was analyzed by Leica imagescope software (ImageScope, RRID: SCR_014311).

### 2.12 Statistics

All statistical analyses were performed using graphpad prism software 8.0 (RRID: SCR_002798) (GraphPad Software Inc., San Diego, California, USA) and spss 18.0 software (RRID: SCR_002865) (IBM SPSS Statistics Inc., Chicago, Illinois, China). Most of the data were analyzed by a two‐tailed Student's *t*‐test or one‐way ANOVA with Dunnett's test for pairwise comparisons. Tumor volumes were analyzed by a two‐tailed paired Student's *t*‐test. Correlations were analyzed by the Pearson's test. The expression of CDKN1A in paired cancer and adjacent non‐neoplastic tissue was analyzed by a two‐tailed paired Student's *t*‐test. Phase‐contrast and fluorescence pictures were taken with a Nikon Eclipse A1 microscope (Tokyo, Japan). The IHC staining intensity was analyzed by imagescope software (ImageScope, RRID: SCR_014311). The Wilcoxon signed‐rank test was used to compare paired non‐normally distributed data. Bars show the mean ± SD or SEM of three independent repeated experiments. Significant differences were accepted if the *P*‐value was < 0.05.

## 3 Results

### 3.1 Tumor cell lines harboring *K‐RAS* mutation are more sensitive to M1 virus than those without *K‐RAS* mutation

**Fig. 1:**
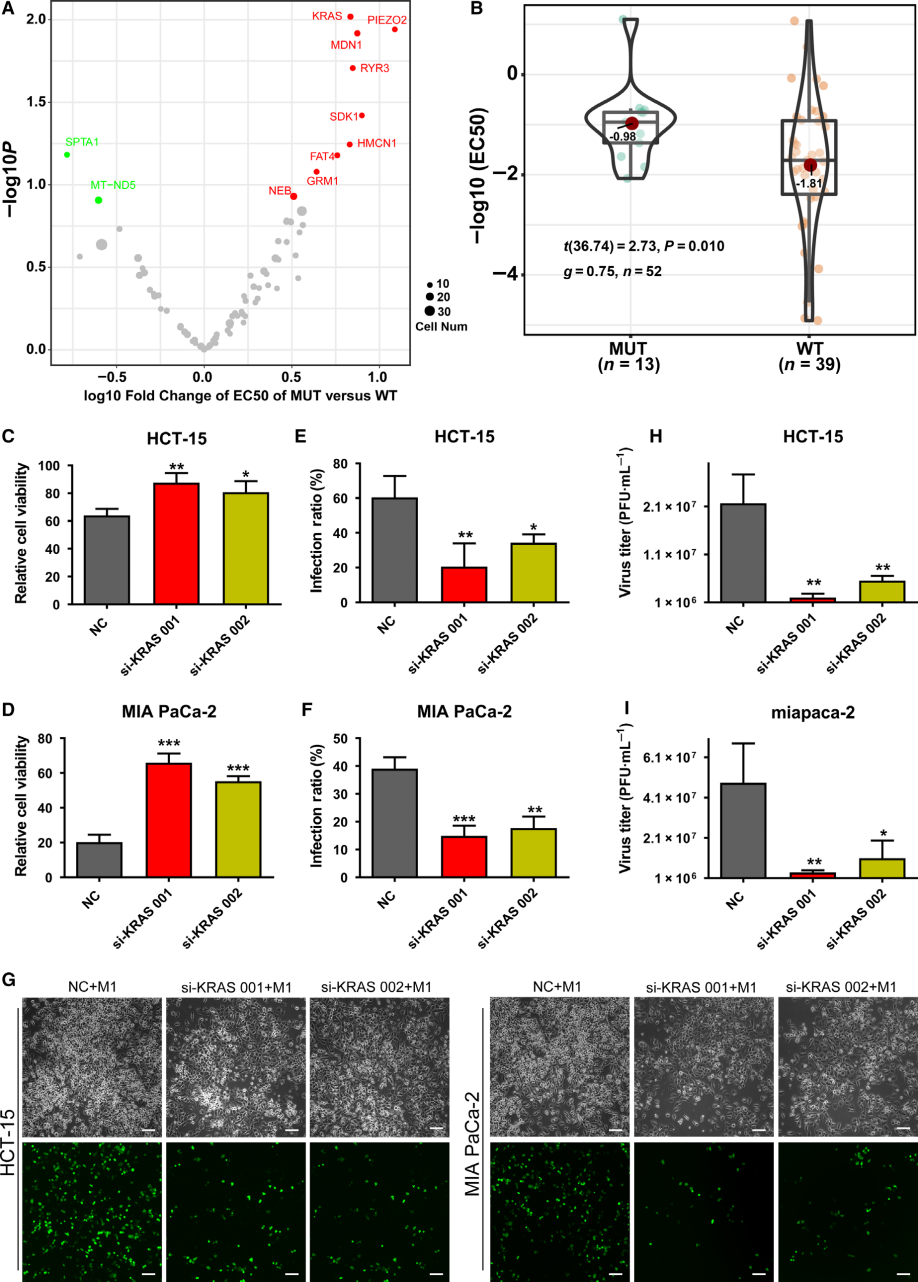
Tumor cells harboring mutations in *K‐RAS* were more sensitive to M1 virus than those not harboring mutations in *K‐RAS*. (A) Volcano plot showing the relationship between the oncolytic effect of M1 virus and oncogenic mutations in 52 tumor cell lines. The *x*‐axis shows the log fold change of the oncolytic effect (represented by EC50 values) in *K‐RAS* mutant cells versus *K‐RAS* wild‐type cells. The EC50 values of M1 were analyzed as below: 52 tumor cell lines were treated with different MOI of M1 virus (0, 0.001, 0.1, 1, 10, and 100 (100 MOI for resistant cell lines such as HCC827, HEL, ME180, Reh, and SiHa)) for 48 h, the cell‐killing percentage was detected by MTT. The dose–response curve of multi‐MOI of M1 virus was fitted with nonlinear regression in each cell line, and EC50 (viral dose to kill 50% cancer cells) was calculated. The *y*‐axis shows the log_10_ *P* values analyzed by the chi‐square test; *n* = 3. (B) Violin figure showing the EC50 of M1 virus in *K‐RAS* wild‐type and mutant cancer cells; *n* = 3. MUT: mutation. Statistical analysis was performed by two‐tailed Student's *t*‐test. (C,D) The HCT‐15 and MIA PaCa‐2 cell lines were treated with siRNAs targeting K‐RAS for 48 h, M1 virus (MOI = 1 pfu/cell) was added for another 60 h, and the viability was detected by MTT; *n* = 3. Statistical analysis was performed by one‐way ANOVA with Dunnett's test for pairwise comparisons. (E‐I) The HCT‐15 and MIA PaCa‐2 cell lines were treated with siRNAs targeting K‐RAS for 48 h, M1 virus (MOI = 1 pfu/cell) was added for another 24 h, and the infection rate of M1 virus (GFP percentage) was detected by flow cytometry (E,F). Phase‐contrast and fluorescence pictures are shown (G). Titer of M1 virus was detected by TCID50 method (H,I). *n* = 3. Statistical analysis was performed by one‐way ANOVA with Dunnett's test for pairwise comparisons. The pictures show one representative result from three similar experimental replicates. Scale bar, 100 μm. Error bars represent the mean ± SD obtained from three independent experiments. *P < 0.05, **P < 0.01, ***P < 0.001. See also in Fig. S1, Tables S1, and S2. For TCID50 assay, the starting cell numbers of compared group are the same.

To investigate the relationship between the selectivity of M1 virus and oncogenic signals in tumors, we analyzed the relationship between the oncolytic effect (represented by values of EC50) of M1 virus (Table S1) and cellular oncogenic mutations in 52 tumor cell lines originating from various types of tissue. The oncogenic mutation data were retrieved from the Cancer Cell Line Encyclopedia (CCLE) database [30], and all of the mutations in the 52 cell lines are listed in Table S2. By analyzing the EC50 values of M1 virus in these cell lines with or without oncogenic mutations, we found that the *K‐RAS*, *MDN1*, *RYR3,* and *PIEZO2* genes are frequently mutated in the cell lines with higher sensitivity to M1 virus (Fig. 1A). Of the genes listed, *K‐RAS* is the most notable and undruggable target, and further statistical analysis confirmed that M1 virus showed lower EC50 values which represent better antitumor effects in the cell lines with *K‐RAS* mutation than those with wild‐type *K‐RAS* (Fig. 1B and Table S1).

To test whether mutant *K‐RAS* indeed affects the sensitivity of cancer cells to M1 virus, specific siRNAs to *K‐RAS* were used to knock down the expression *of K‐RAS* in HCT‐15 and MIA PaCa‐2 cell lines (Fig. S1), which harbor the mutant *K‐RAS* (indicated in Table S1). In these cell lines, knockdown of *K‐RAS* inhibited the cell killing by M1 virus (Fig. 1C,D) and suppressed the infection rate of M1 virus as shown by flow cytometry and fluorescence imaging (Fig. 1E‐G). Moreover, knockdown of *K‐RAS* inhibited the replication of M1 virus as shown by viral titer detected by TCID50 method (Fig. 1H–I). Taken together, the results suggest that *K‐RAS* mutation promotes the replication and subsequent oncolytic effect of M1 virus.

### 3.2 RAS/RAF/MEK signaling inhibitors suppress the oncolytic efficiency and gene expression of M1 virus

**Fig. 2:**
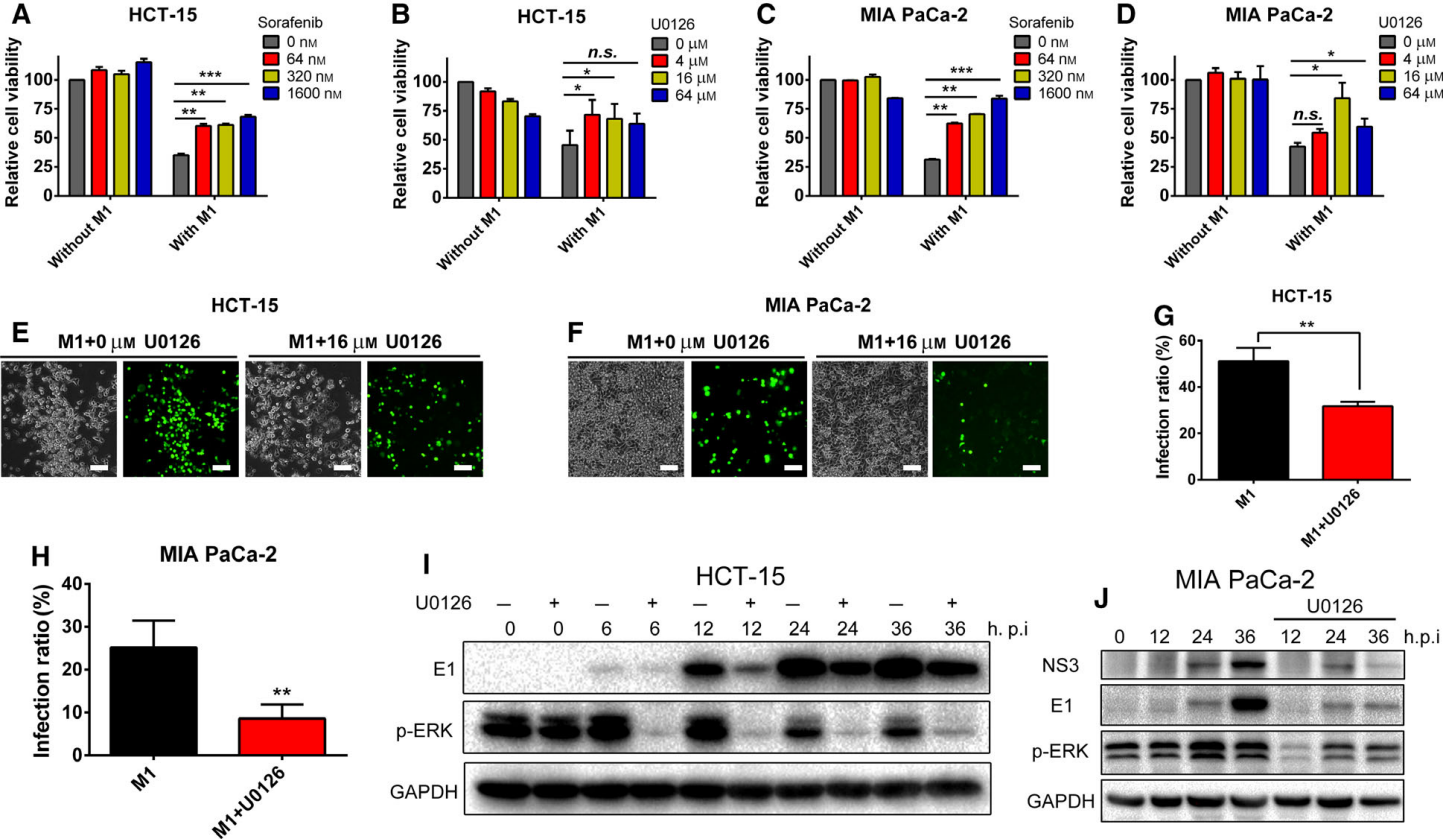
Sorafenib and U0126 inhibited the oncolytic effect and replication of M1 virus. (A‐D) HCT‐15 and MIA PaCa‐2 cell lines were treated with different concentrations of sorafenib or U0126 with or without M1 (MOI = 1 pfu/cell) for 60 h, and cell viability was detected by MTT; *n* = 3. Statistical analysis was performed by one‐way ANOVA with Dunnett's test for pairwise comparisons. (E,F) HCT‐15 and MIA PaCa‐2 cell lines were treated with control, M1 (MOI = 1 pfu/cell), U0126 (16 μm), or M1 (MOI = 1 pfu/cell) plus U0126 (16 μm) for 24 h, and phase‐contrast and fluorescence pictures are shown. The results show one representative result from three similar experimental replicates. Scale bar, 100 μm. (G,H) HCT‐15 and MIA PaCa‐2 cells were treated with M1 (MOI = 1 pfu/cell) and M1 (MOI = 1 pfu/cell) plus U0126 (16 μm) for 24 h, and the infection rate of M1 virus (GFP percentage) was detected by flow cytometry; *n* = 3. Statistical analysis was performed by two‐tailed Student's *t*‐test. (I,J) HCT‐15 and MIA PaCa‐2 cells were treated with control, M1 (MOI = 1 pfu/cell), U0126 (16 μm), or M1 (MOI = 1 pfu/cell) plus U0126 (16 μm) for the indicated times, and the levels of E1, NS3, and p‐ERK were detected by western blot. The results show one representative result from three similar experimental replicates. Error bars represent the mean ± SD obtained from three independent experiments. n.s., not significant; *P < 0.05, **P < 0.01, ***P < 0.001. See also in Fig. S2.

*RAS* mutation results in the activation of the RAS/RAF/MEK pathway, so we investigated whether inhibitors of the RAS/RAF/MEK pathway affect the oncolytic efficiency and gene expression of M1 virus in tumor cells. Sorafenib is an approved drug for the treatment of different types of cancer that has been reported to preferably inhibit the activity of RAF [31], while U0126 selectively inhibits MEK1/2 [32]. In the pancreatic carcinoma cell line MIA PaCa‐2 and colorectal carcinoma cell line HCT‐15, both sorafenib and U0126 modestly but significantly inhibited the oncolytic effect of M1 (Fig. 2A–D). Cobimetinib and trametinib, two other MEK inhibitors approved by the FDA, also modestly but significantly inhibited the oncolytic effect of M1 virus (Fig. S2A‐D). We previously reported that the cancer targeting and killing properties of M1 virus depend on the replication of the virus in cancer cells [23], so we used M1 virus engineered to express the reporter protein GFP (M1‐GFP) to trace the gene expression of the virus in tumor cells. Phase‐contrast and fluorescence imaging showed that U0126 suppressed the reporter gene expression and M1‐induced cytopathic effects (Fig. 2E,F). Cytometry analysis consistently proved that the M1 virus infection rate was modestly but significantly inhibited by U0126 treatment (Fig. 2G,H). Furthermore, the expression of viral proteins E1 and NS3 decreased significantly following U0126 treatment, which effectively inhibited the phosphorylation of ERK (Fig. 2I,J). In conclusion, RAS/RAF/MEK signaling promotes the oncolytic effect of M1 virus by upregulating viral gene expression, which suggests the acceleration of viral replication.

### 3.3 Inhibition of the RAS/RAF/MEK pathway upregulates the expression of antiviral signaling pathway members

**Fig. 3:**
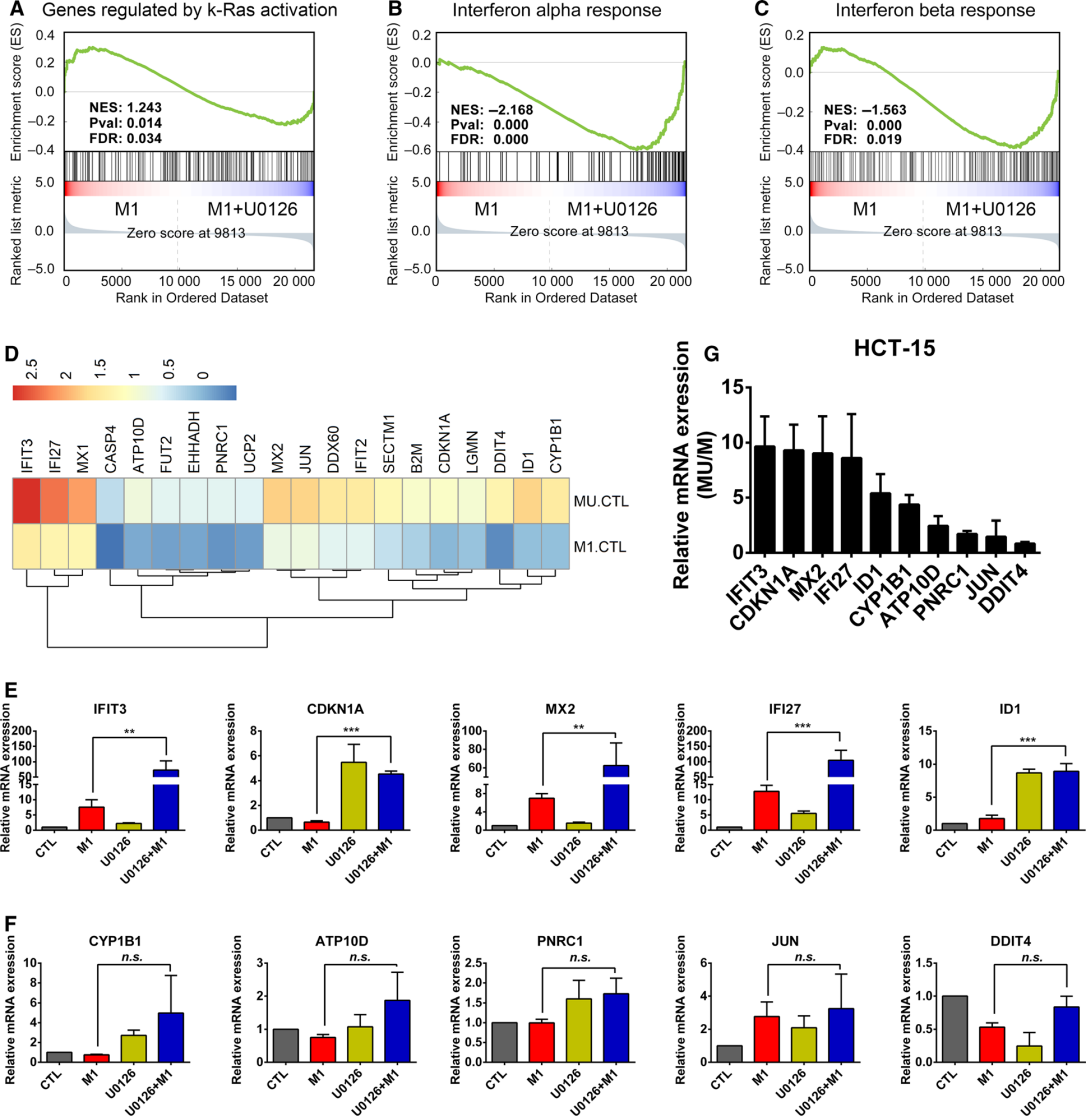
U0126 inhibited RAS signaling and upregulated the expression of antiviral genes. (A‐C) GSEA of the RAS‐regulated gene set (A), IFN alpha response gene set (B), and IFN beta gene set (C) after M1 (MOI = 1 pfu/cell) and M1 (MOI = 1 pfu/cell) plus U0126 (16 μm) treatment of the HCT‐15 cell line for 24 h. Values of NES, *p*, and FDR are shown in each box. (D) The heatmap of the top 20 IRGs upregulated in the U0126 plus M1‐treated HCT 15 cells compared with M1 (MU/M)‐treated HCT 15 cells is shown. (E‐F) The HCT‐15 cell line was treated with control, M1 (MOI = 1 pfu/cell), U0126 (16 μm), or M1 (MOI = 1 pfu/cell) plus U0126 (16 μm) for 24 h, and the relative expression of the top 10 IRGs (including *IFIT3*, *CDKN1A*, *MX2*, *IFI27*, *ID1*, *CYP1B1*, *ATP10D*, *PNRC1*, *JUN*, and *DDIT4*) in (D) was detected by qPCR; *n* = 3. Statistical analysis was performed by one‐way ANOVA with Dunnett's test for pairwise comparisons. (G) Summary of the top 10 IRGs treated with U0126 plus M1 compared with M1 (MU/M). Error bars represent the mean ± SD obtained from three independent experiments. CTL, control; MU, M1 + U0126. n.s., not significant; *P < 0.05, **P < 0.01, ***P < 0.001. See also Tables S3–S5.

To identify the mechanism by which RAS/RAF/MEK signaling promotes the replication of M1, gene expression profiling was performed in the HCT‐15 cell line under vehicle, U0126, M1, or M1 plus U0126 treatment conditions. Gene set enrichment analysis (GSEA) proved that U0126 effectively downregulated the RAS signaling pathway (Fig. 3A and Table S3). It is well known that the promotion of viral replication by oncogenic signals is due to crosstalk of them with antiviral pathways, which consist mainly of interferon (IFN) signaling [7]. Among the three classes of IFNs, type I IFNs are known to be essential for mounting a robust host response against viral infection [33]. We hypothesized that U0126 might upregulate antiviral IFN pathway activity to inhibit the replication and oncolytic effect of M1 virus.

Gene set enrichment analysis revealed that U0126 strongly upregulated the IFN‐α and IFN‐β response pathways after M1 virus infection (Fig. 3B,C and Tables S4–S5). To identify the key factors upregulated by U0126 to inhibit the replication and oncolytic effect of M1 virus, we focused on the expression of 317 IFN‐regulated genes (IRGs), which were identified to be crucial antiviral effectors for alphavirus M1 [23]. The heatmap of the top 20 IRGs upregulated by U0126 plus M1 compared with M1 (MU/M) is shown in Fig. 3D. In addition, qPCR was used to verify the expression of the top 10 IRGs in HCT‐15 cell line. We found that the expression of *IFIT3*, *CDKN1A*, *MX2*, *IFI27,* and *ID1* was significantly increased in the M1 plus U0126 group compared with the M1 treatment group (Fig. 3E,F). When we ranked the fold change between the M1 plus U0126 group and the M1 group (MU/M), these five genes comprised the top five (Fig. 3G). Taken together, these results indicate that the inhibition of RAS signaling by U0126 upregulates the IFN‐mediated innate immune response of cancer cells, which may result in the attenuation of the replication and oncolytic effect of M1 virus.

### 3.4 CDKN1A is a key antiviral factor downstream of RAS signaling that inhibits the replication of M1 virus

**Fig. 4:**
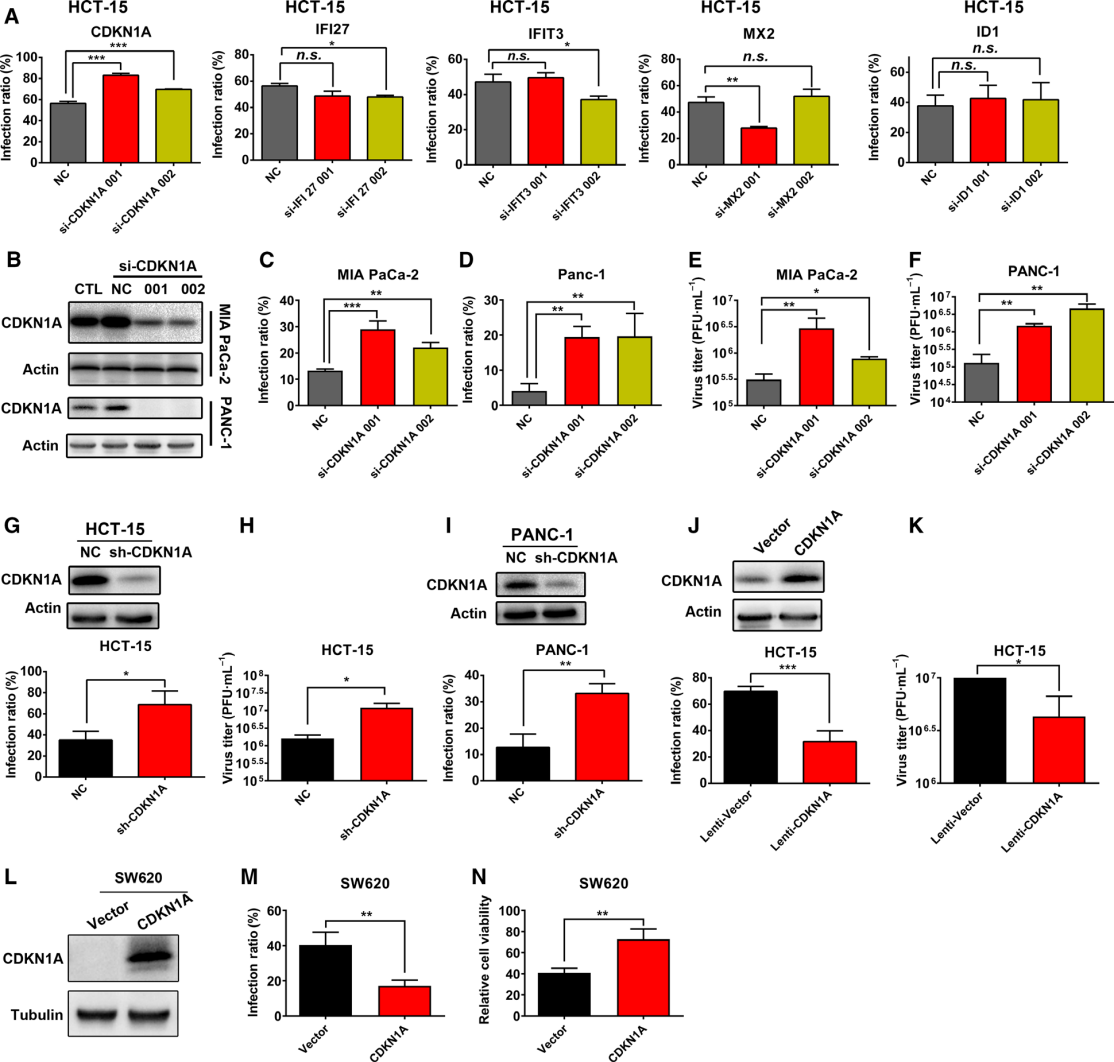
CDKN1A was the key IRG that inhibited the replication of M1 virus. (A) The HCT‐15 cell line was treated with siRNAs targeting IFIT3, CDKN1A, MX2, IFI27, and ID1 for 48 h, M1 virus (MOI = 1 pfu/cell) was added for another 24 h, and the infection rate of M1 virus (GFP percentage) was detected by flow cytometry; *n* = 3. Statistical analysis was performed by one‐way ANOVA with Dunnett's test for pairwise comparisons. (B) The knockdown efficiency of siRNAs (48 h) targeting CDKN1A in MIA PaCa‐2 and PANC‐1 cell lines detected by western blot. (C‐F) MIA PaCa‐2 and PANC‐1 cell lines were treated with siRNAs against CDKN1A for 48 h, M1 virus (MOI = 1 pfu/cell) was added for another 24 h, and the infection rate (C,D) and titer (E,F) of M1 virus were detected by flow cytometry and the TCID50; *n* = 3. Statistical analysis was performed by one‐way ANOVA with Dunnett's test for pairwise comparisons. (G‐I) The infection rate and titer of M1 virus in HCT‐15 and PANC‐1 cell lines treated with or without shCDKN1A were detected by flow cytometry and the TCID50. The efficiency of shCDKN1A was detected by western blot; *n* = 3. Statistical analysis was performed by two‐tailed Student's *t*‐test. (J,K) The infection rates and titers of M1 virus in the HCT‐15 cells treated with or without CDKN1A were detected by flow cytometry and the TCID50. The overexpression of CDKN1A by lentivirus was detected by western blot; *n* = 3. Statistical analysis was performed by two‐tailed Student's *t*‐test. (L) SW620 cell line was transfected by CDKN1A vector or control vector; the overexpression of CDKN1A was verified by western blot. (M‐N) SW620 cells transfected with or without CDKN1A vector were treated with M1 virus (MOI = 1 pfu/cell) for 24 h, infection rate of M1 was detected by flow cytometry (M). Sixty hours later, cell viability was detected by MTT method (N). *n* = 3. Statistical analysis was performed by two‐tailed Student's *t*‐test. Error bars represent the mean ± SD obtained from three independent experiments. n.s., not significant; *P < 0.05, **P < 0.01, ***P < 0.001. See also Figs S3 and S4. For TCID50 assay, the starting cell numbers of compared group are the same.

To further identify the specific IRG that inhibits M1 viral infection, we used siRNAs to knock down the five genes identified above in the HCT‐15 cell line, including *CDKN1A*, *IFI27*, *IFIT3*, *MX2,* and *ID1*. The siRNA effectively knocked down the expression of these genes (Fig. S3), and only the knockdown of CDKN1A significantly increased the infection of M1 virus (Fig. 4A), indicating that CDKN1A might be a key factor. CDKN1A, also known as p21, is a universal cell cycle inhibitor directly controlled by p53 and p53‐independent pathways [34]. To further elucidate the antiviral function of CDKN1A, we knocked down the expression of CDKN1A in two more pancreatic carcinoma cell lines, MIA PaCa‐2 and PANC‐1 (Fig. 4B). Knocking down CDKN1A increased both the M1 infection and viral titer in these cell lines (Fig. 4C–F). Further knockout of CDKN1A in HCT‐15 and PANC‐1 cell lines by shRNA also increased the infection and titer of M1 virus (Fig. 4G–I). Further, the lentivirus‐mediated overexpression of CDKN1A notably reduced the M1 virus infection and titer (Fig. 4J,K). Moreover, the overexpression of CDKN1A in SW620 colorectal carcinoma cell line which is CDKN1A‐defective (Fig. S4) decreased the infection rate of M1 virus and attenuated the subsequent cell killing of M1 (Fig. 4L‐N). In summary, these loss‐ and gain‐of‐function experiments demonstrated that CDKN1A is the key IRG that is suppressed by RAS/RAF/MEK signaling to promote the replication of M1 virus.

**Fig. 5:**
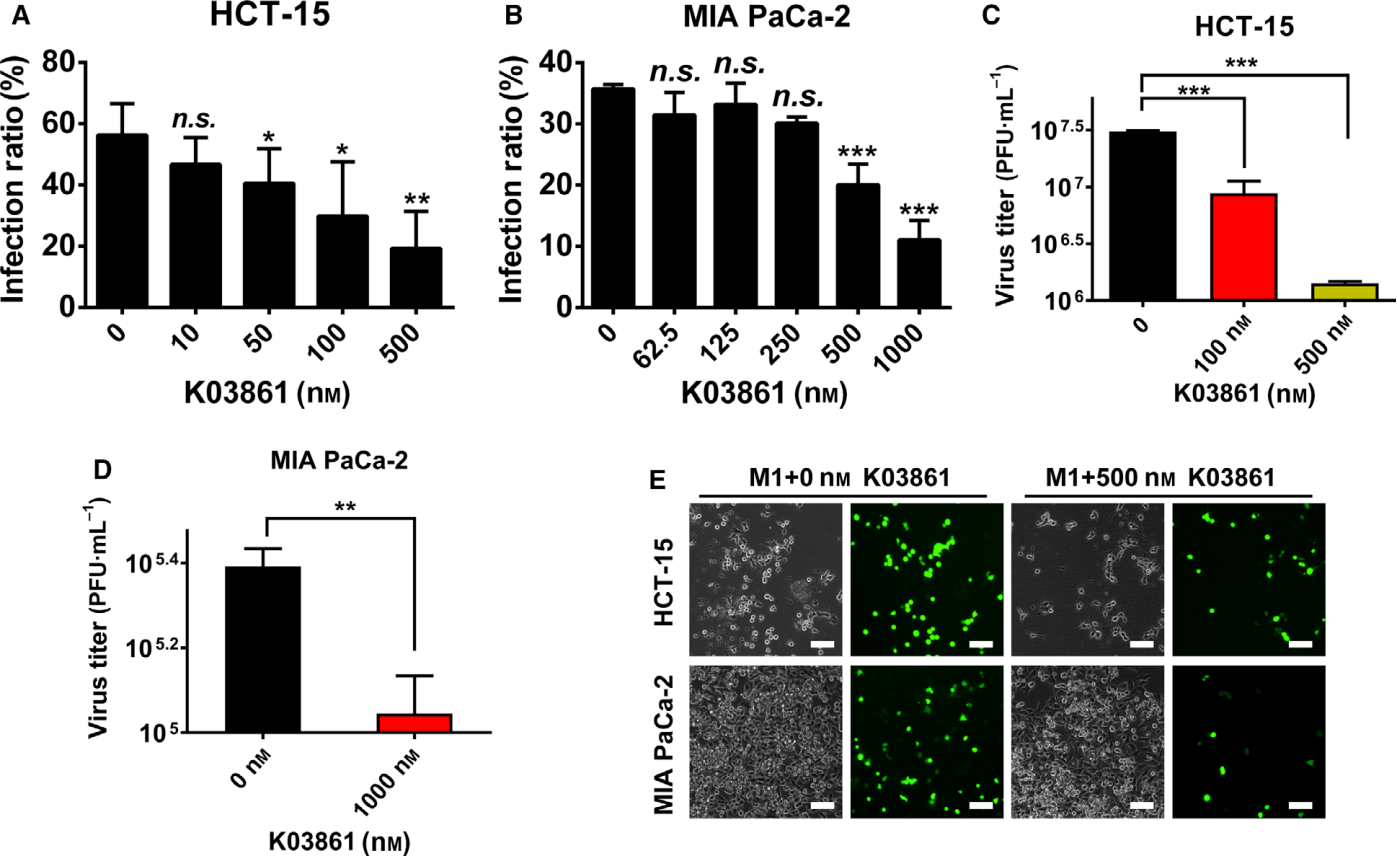
The CDK2 inhibitor K03861 inhibited the replication of M1 virus. (A‐E) HCT‐15 and MIA PaCa‐2 cells were treated with different concentrations of K03861 with or without M1 virus (MOI = 1 pfu/cell) for 24 h; then, the infection rate (A and B) was detected by flow cytometry, the titer was detected by TCID50 (C and D), and phase‐contrast and fluorescence pictures were taken (E). Statistical analysis was performed by two‐tailed Student's *t‐*test or one‐way ANOVA with Dunnett's test for pairwise comparisons. Error bars represent the mean ± SD obtained from three independent experiments. n.s., not significant; *P < 0.05, **P < 0.01, ***P < 0.001. For TCID50 assay, the starting cell numbers of compared group are the same.

CDKN1A is a tumor suppressor that mainly functions as a cell cycle checkpoint by binding to CDK1, Cyclin‐dependent kinase 2 (CDK2), or the CDK4/6 complex [35]. We investigated whether the cell cycle plays an important role in the replication of M1 virus by inhibiting the activity of CDK2, a primary factor mediating the cell cycle inhibition activity of CDKN1A. K03861, an inhibitor of CDK2, modestly but significantly decreased the infection rate of M1 virus in a concentration‐dependent manner in HCT‐15 and MIA PaCa‐2 cell lines (Fig. 5A,B), and titer of M1 virus was subsequently significantly inhibited by K03861 in these cell lines (Fig. 5C,D), phase‐contrast and fluorescence imaging also showed the inhibition of M1 viral infection by K03861 (Fig. 5E). The results indicate that CDKN1A inhibits the replication of M1 virus by suppressing the cell cycle.

### 3.5 **Knockdown of CDKN1A enhances the oncolytic effect of M1 virus** *in vivo*

**Fig. 6:**
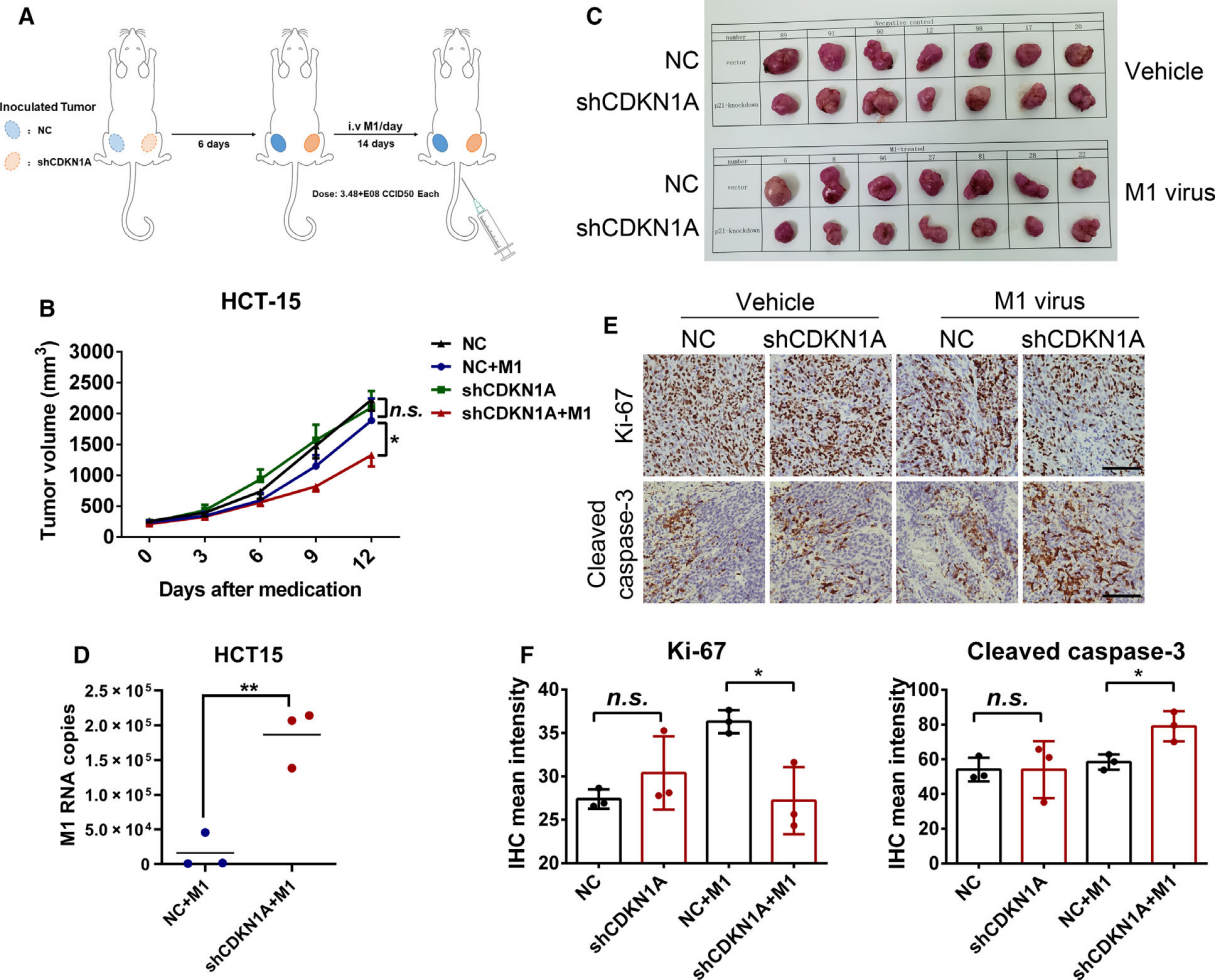
Knockdown of CDKN1A by shRNA enhanced the oncolytic effect of M1 virus in HCT‐15 xenograft tumors. (A) Schematic of the *in vivo* experiment. In brief, HCT‐15‐NC and HCT‐15‐shCDKN1A cells were inoculated in each hind flank of the nude mice. Six days later, tumors were visible, and M1 virus was injected intravenously for 14 days. Tumors were measured every other day, and the tumor volume was calculated by the formula (length × width^2^)/2. (B) Growth curve of the tumors in each group; *n* = 7. Error bars represent the mean ± SEM. Statistical analysis was performed by two‐tailed paired Student's *t*‐test. (C) At the endpoint, mice were anesthetized and sacrificed, and tumors were subsequently dissected and photographed. (D) Three days after the first medication, total RNA in tumors was extracted and viral copy numbers were detected by TaqMan qRT‐PCR. *n* = 3; statistical analysis was performed by two‐tailed paired Student's *t*‐test. (E) At the endpoint, the expression of Cl‐casp3 and Ki‐67 in tumors was detected by IHC. Scale bars, 100 μm. (F), Statistical analysis of Ki‐67 and Cl‐casp3 IHC intensity; *n* = 3; error bars represent the mean ± SD. Statistical analysis was performed by two‐tailed paired Student's *t*‐test. n.s., not significant; *P < 0.05, **P < 0.01.

To further validate whether the suppression of CDKN1A enhances the oncolytic effect of M1 virus *in vivo*, we established an HCT‐15 subcutaneous xenograft model in nude mice. HCT‐15‐NC cells and HCT‐15‐shCDKN1A cells were implanted in the left and right hind flanks of mice, and M1 virus was injected through the caudal vein (Fig. 6A). Compared with that of HCT‐15‐NC tumors, the growth of HCT‐15‐shCDKN1A tumors treated with M1 virus was modestly but significantly inhibited (Fig. 6B), and the tumor size in the HCT‐15‐shCDKN1A group treated with M1 virus was smaller than that in the other three groups (Fig. 6C). Consistent with the cellular results, M1 viral copy number in HCT‐15‐shCDKN1A tumors was increased compared with HCT‐15‐NC tumors (Fig. 6D). Furthermore, immunohistochemical (IHC) staining was performed on tumor tissues to detect the expression of Ki‐67 and Cl‐casp3 to measure the proliferation and apoptosis of tumor cells. We observed that Ki‐67 expression was significantly downregulated and Cl‐casp3 expression was coordinately upregulated in the M1 virus‐treated HCT‐15‐shCDKN1A tumors (Fig. 6E,F). These results further support the hypothesis that CDKN1A acts as an antiviral factor to inhibit the oncolytic effect of M1 virus *in vivo*.

### 3.6 CDKN1A deficiency is a biomarker for M1 therapy

**Fig. 7:**
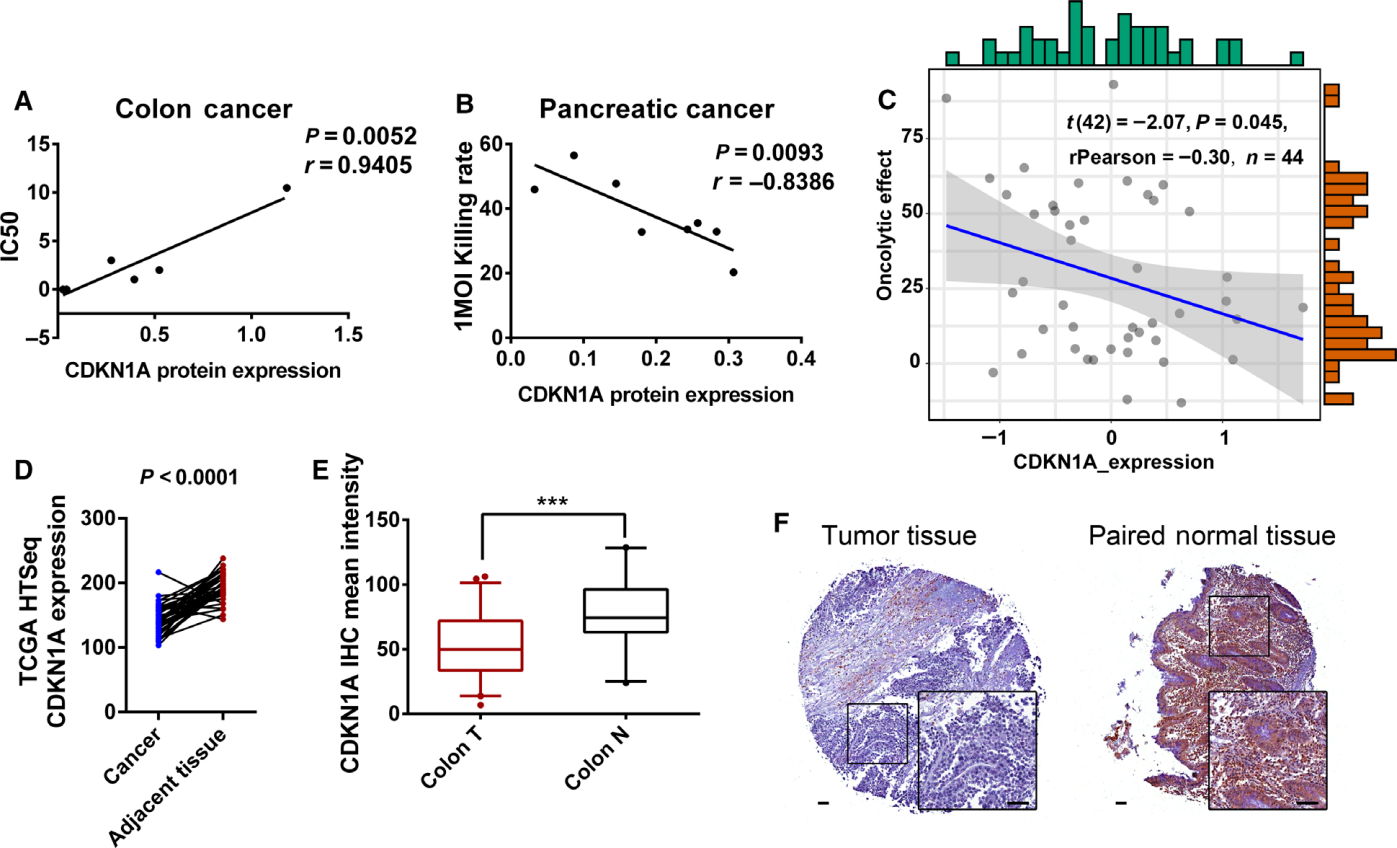
Expression of CDKN1A negatively correlated with the oncolytic effect of M1 virus and served as a biomarker for M1. (A‐B) The correlation between the oncolytic effect of M1 virus and the protein expression of CDKN1A in colorectal cancer cell lines (A) and pancreatic cancer cell lines (B). The protein level of CDKN1A was detected by western blot (Fig. S4), and the oncolytic effect of M1 virus is shown in Table S6. Statistical analysis was performed by the Pearson correlation test. (C) The correlation between the oncolytic effect of M1 virus and protein expression of CDKN1A in 44 tumor cell lines. The oncolytic effect of M1 was indicated by the cell‐killing percentage in the 44 tumor cell lines treated with M1 virus (MOI = 10 pfu/cell) for 48 h and detected by MTT; *n* = 3. The *x*‐axis shows the protein expression of CDKN1A measured by RPPA in the CCLE data. Statistical analysis was performed by the Pearson correlation test. (D) HTSeq count showing the mRNA expression of CDKN1A in tumor and adjacent non‐neoplastic tissue from colon cancer patients in the TCGA data; the percentage of patients showing lower expression of CDKN1A in tumor tissue than that in adjacent non‐neoplastic tissue was calculated. Blue dots represent tumor tissues, red dots represent paired adjacent non‐neoplastic tissues, and black lines connect paired tumor and adjacent non‐neoplastic tissue from the same patient. *n* = 38. Statistical analysis was performed by two‐tailed paired Student's *t*‐test. (E) IHC staining intensity of CDKN1A expression in TMAs containing paired tumor and adjacent non‐neoplastic clinical specimens from 143 colon cancer patients. The IHC level is presented as the mean staining intensity calculated using imagescope software (Leica); the percentage of patients showing lower expression of CDKN1A in tumor tissue than that in adjacent non‐neoplastic tissue was calculated. Statistical analysis was performed by two‐tailed paired Student's *t*‐test. (F) Representative pictures of the IHC in (E). Scale bars, 50 μm. *P < 0.05, **P < 0.01, ***P < 0.001. See also Fig. S4 and Tables S6 and S7.

The above CDKN1A loss‐ and gain‐of‐function experiments indicated that M1 may specifically target cancer cells deficient in CDKN1A. Therefore, we detected the protein expression of CDKN1A in pancreatic carcinoma and colorectal carcinoma cell lines, which have been reported to frequently harbor *K‐RAS* mutations, and analyzed the correlation between the cell‐killing effect of M1 virus and CDKN1A expression. In both pancreatic and colorectal cancer cell lines, there is a significant negative correlation between CDKN1A expression and the killing capacity of M1 virus, which means that the lower the protein expression of CDKN1A is, the greater the cell‐killing effect of M1 virus is (Fig. 7A,B, the protein expression and cell‐killing effect of M1 are shown in Fig. S4 and Table S6). Furthermore, the relationship between the oncolytic effect of M1 and the expression of CDKN1A was also analyzed in 44 tumor cell lines, and CDKN1A expression was measured via reversed‐phase protein array (RPPA) in the CCLE database. The expression of CDKN1A negatively correlated with the oncolytic effect of M1 in 44 tumor cell lines (Fig. 7C, Table S7). These results suggest that the protein expression of CDKN1A might predict the killing effect of M1 virus.

To elucidate the potential of M1 virus personalized therapy by detecting CDKN1A deficiency, we compared the expression of CDKN1A in both tumor and adjacent non‐neoplastic tissue specimens from 38 colon cancer patients in the TCGA database [36]. The expression of CDKN1A in tumor tissues was lower than that in adjacent non‐neoplastic tissues in over 90% (35/38) of patients (Fig. 7D). Furthermore, we performed IHC on two tissue microarrays (TMAs) containing paired tumor and adjacent non‐neoplastic clinical specimens from 143 colon cancer patients. CDKN1A expression was represented by the mean staining intensity, calculated by imagescope software (Leica, Weztlar, Germany). The expression of CDKN1A in tumor tissues was lower than that in adjacent non‐neoplastic tissues in 58% (83/143) of patients (Fig. 7E,F). Both the database and our experiments strongly suggest that the expression of CDKN1A was frequently deficient in colorectal cancers, which implies the application of M1 virus.

## 4 Discussion

In this paper, we report that the oncogenic RAS/RAF/MEK pathway promotes the replication and oncolytic effect of M1 virus by inhibiting the expression of the key antiviral factor CDKN1A. More importantly, cancer cell lines with lower expression levels of CDKN1A showed greater sensitivity to M1 virus than those with higher levels, and the deficiency of CDKN1A is a ubiquitous event in colorectal cancer patients. Our data suggest that CDKN1A is a suitable biomarker for M1 virus therapy. Our report provides a candidate research program and working model to screen and identify oncogenic pathways as well as biomarkers that other oncolytic viruses utilize to facilitate their replication.

Despite more than three decades of intensive effort, no effective pharmacologic inhibitors of the RAS oncoproteins have reached the clinic, promoting the widely held perception that RAS proteins are ‘undruggable’ [37]. According to our results, M1 virus might offer a renewed hope for the treatment of cancer with abnormal *RAS* activation, making *RAS*‐driven tumors ‘druggable’. Among all cancer‐driven genes, *RAS* is the most frequently mutated oncogene family in human cancers. The top four cancer types harbor *RAS* mutation including pancreatic ductal adenocarcinoma (97.7%), colorectal adenocarcinoma (52.2%), multiple myeloma (42.6%), and lung adenocarcinoma (32.2%) [37]. The high percentage of *RAS* mutation in tumors indicates that M1 virus might be effective in a large proportion of cancer patients.

The hypothesis that oncolytic viruses exploit the deficiencies of antiviral pathways and oncogenic signaling between tumor and normal cells to selectively target and kill cancer cells has been strongly proven. The RAS pathway is a typical oncogenic pathway reported to facilitate the replication of various oncolytic viruses. However, to date, no clinical trials have validated *RAS* mutations as a biomarker for any oncolytic virus, which may be due to the upregulation of other compensatory pathways downstream of RAS that control the expression of antiviral genes in patients; thus, identifying the antiviral genes regulated by the RAS pathway as biomarkers for oncolytic viruses is urgently required. The expression of components of the IFN pathway, such as PKR and IRF1, has been reported to be downregulated by the RAS pathway. However, the expression of these genes has not been proven to be substantially decreased in tumors. Here, we found a new antiviral gene, CDKN1A, downstream of the RAS pathway to suppress the replication and oncolytic effect of M1 virus, and it has been reported to be a tumor suppressor. Our data clearly support the hypothesis that deficiency in both tumor suppressor and antiviral pathways supports the tumor selection mechanism of oncolytic viruses and provides a new biomarker for accurate oncolytic virotherapy.

CDKN1A is a universal cell cycle inhibitor directly controlled by p53 and p53‐independent pathways, and the loss of CDKN1A causes carcinogenesis by inducing growth arrest, regulating the expression of genes associated with senescence, and protecting cells from apoptosis [34, 35, 38]. Consistently, our data prove that CDKN1A expression is consistently deficient in colon cancer, further supporting the reports that CDKN1A is a tumor suppressor. In this study, we demonstrate that CDKN1A inhibits the replication of M1, whereas the inhibition of CDKN1A promotes the replication and oncolytic effect of M1 virus. In tumors with low CKDN1A expression, the oncolytic effect of M1 virus is stronger than that in tumors with high CDKN1A expression. We hypothesize that CDKN1A controls the cell cycle, but viral replication needs increased amounts of material and energy, so in cancer cells without CDKN1A to control proliferation, the proliferation rate increases and provides sufficient cellular resources for the synthesis and assembly of new viral particles.

Genetic heterogeneity represents one of the most significant hallmarks of cancer, indicating that universal treatment for all patients is problematic and that personalized medicine is required for cancer therapy. The discovery of biomarkers in tumors provides a potential strategy to solve this problem [39]. The detection of biomarkers before treatment with an anticancer drug decreases the chance of a patient receiving an ineffective medication and largely increases the efficiency of the drug. In 2017, the PD‐1 antibody pembrolizumab was approved by the FDA for patients with microsatellite instability‐high and different mismatch repair [40]. It is the first anticancer strategy that is distinguished by a biomarker but not the organ origin of the tumor. In this study, we found that the oncolytic effect of M1 virus negatively correlated with the expression of CDKN1A in tumor cells. CDKN1A serves as a tumor suppressor and is deficient in various cancer types in addition to colorectal carcinoma [41], which indicates that CDKN1A expression may serve as an M1 virus biomarker in pan‐cancer types.

Sorafenib is the first oral multikinase inhibitor that targets RAF. It was first approved by the FDA for the treatment of advanced renal cell carcinoma in 2005 [31]. Subsequently, it was approved for other indications, including hepatocellular carcinoma and radioactive iodine‐refractory differentiated thyroid cancer [42]. We have previously reported that M1 virus has a natural tropism for hepatocellular carcinoma and multiple kinds of cancers [23]. Moreover, we have identified various anticancer chemicals in clinical use or in the clinical trial stage, such as VCP inhibitors [27], DNK‐PK inhibitors [28], Smac mimetics [24], and Bcl‐XL inhibitors [26], can sensitize tumors to M1 virus. Here, we report that sorafenib inhibits the replication and oncolytic effect of M1 virus. These results suggest that in the future, the combination of M1 virus and sorafenib should be avoided in the clinic.

## 5 Conclusions

In summary, our research shows that tumors involving RAS signaling harbor a natural vulnerability to oncolytic M1 virus and illustrate that CDKN1A is the key downstream antiviral factor to predict the oncolytic efficacy of M1 virus.

## Conflict of interest

JFZ, WCX, ZQL, and MS are employers of Guangzhou Virotech Pharmaceutical Co., Ltd. (GVP) and performed the cellular and animal experiments. The present research is not funded by GVP. The authors declare no conflict of interest.

## Author contributions

JC, JKL, and GMY contributed to the experimental design and data analysis. JC, JKL, and MS contributed to manuscript writing and editing. XCL contributed to the data analysis of the expression profile data. KYL, WC, LG, JFZ, WCX, ZQL, and CWH contributed to the cellular and animal experiments. YL contributed to the IHC experiments and IHC data analysis. WBZ and JH contributed to the animal experiments.

## Supporting information


**Table S1.** Oncolytic effect of M1 virus and status in k‐Ras in 52 tumor cells.
**Table S2.** The mutation status of whole genome in 52 tumor cells.
**Table S3.** Genes regulated by *k‐Ras* activation in expression profile database.
**Table S4.** Interferon alpha response genes in expression profile database.
**Table S5.** Interferon beta response genes in expression profile database.
**Table S6.** Oncolytic effect of M1 virus in colon cancer cell lines and pancreatic cancer cell lines.
**Table S7.** Oncolytic effect of M1 virus and the protein expression of CDKN1A in 44 tumor cells.
**Fig. S1.** The efficiency of siRNAs to *K‐RAS*.
**Fig. S2.** Cobimetinib and Trametinib inhibited the oncolytic effect and replication of M1 virus.
**Fig. S3.** The efficiency of siRNAs.
**Fig. S4.** Protein level of CDKN1A.

## Data Availability

The microarray data were submitted to the Gene Expression Omnibus (GEO) (https://www.ncbi.nlm.nih.gov/geo/). The accession number is GSE134487.
