# Supplementary material for: Tumors driven by RAS signaling harbor a natural vulnerability to oncolytic virus M1
Source: Mol Oncol. 2020 Oct 25;14(12):3153–68. doi: 10.1002/1878-0261.12820 (PMC7718955; doi:10.1002/1878-0261.12820)
Supplement: Supplementary file 1 — Table S1. Oncolytic effect of M1 virus and status in k‐Ras in 52 tumor cells. Table S2. The mutation status of whole genome in 52 tumor cells. Table S3. Genes regulated by k‐Ras activation in expression profile database. Table S4. Interferon alpha response genes in expression profile database. Table S5. Interferon beta response genes in expression profile database. Table S6. Oncolytic effect of M1 virus in colon cancer cell lines and pancreatic cancer cell lines. Table S7. Oncolytic effect of M1 virus and the protein expression of CDKN1A in 44 tumor cells. Fig. S1. The efficiency of siRNAs to K‐RAS. Fig. S2. Cobimetinib and Trametinib inhibited the oncolytic effect and replication of M1 virus. Fig. S3. The efficiency of siRNAs. Fig. S4. Protein level of CDKN1A. [file MOL2-14-3153-s001.zip › mol212820-sup-0003-Supinfo.docx]

**Table S1. Oncolytic effect of M1 virus and status in *k-Ras* in** **52 tumor cells. Refers to Figure 1.**

Table S1 shows detail information of 52 tumor cells. 52 tumor cell lines were treated with different MOI of M1 virus (0, 0.001, 0.1, 1, 10 and 100 (100 MOI for resistant cell lines such as HCC827, HEL, ME180, Reh and SiHa)) for 48 hours, the cell killing percentage was detected by MTT. The dose response curve of multi-MOI of M1 virus was fitted with nonlinear regression in each cell line, and EC50 (viral dose to kill 50% cancer cells) was calculated. The mutation status of *k-ras* in these cells was also listed in the table, 1 indicates mutation and 0 indicates non mutation. The data was shown as an additional excel file with the manuscript.

**Table S2. The mutation status of whole genome in 52 tumor cells. Refers to Figure 1.**

Table S2 shows the mutation status of whole genome in 52 tumor cells. The mutation status of the whole genome was got from the Cancer Cell Line Encyclopedia (CCLE) database. 1 indicates mutation, 0 indicates non mutation, NA inducates non avalible. The data was shown as an additional excel file with the manuscript.

**Figure S1. The efficiency of siRNAs to *K-RAS*.**


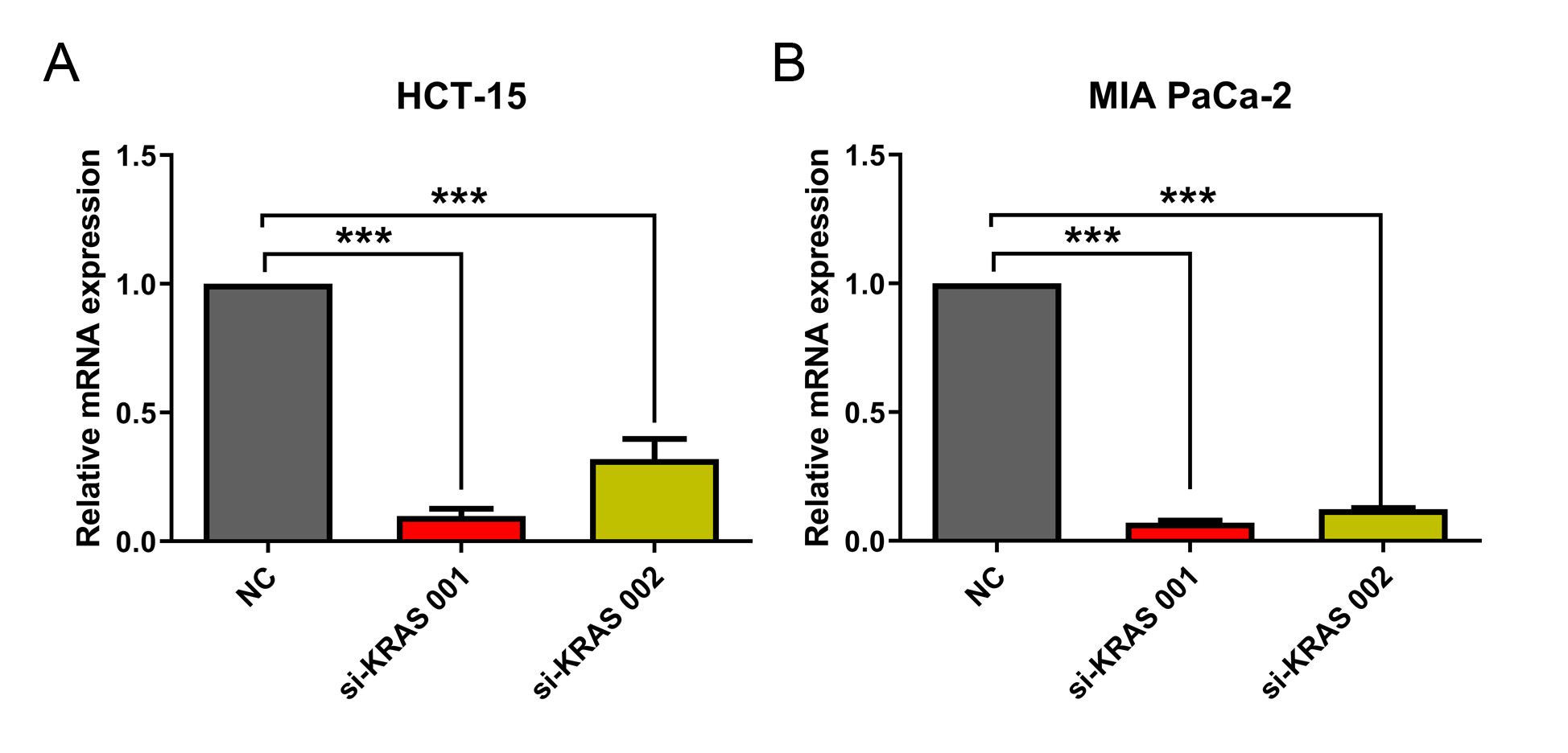


The efficiency of siRNAs to *K-RAS* in HCT-15 and MIA PaCa-2 cell lines. HCT-15 (A) and MIA PaCa-2 (B) cells were treated with siRNAs to K-RAS for 48 hours, relative expression K-RAS was detected by RT-qPCR. Error bars represent mean ± SD obtained from three independent experiments. Statistical analysis was performed by one way ANOVA with Dunnett’s tests for pairwise comparisons. **p*<0.05, ** *p*<0.01, *** *p*<0.001.

**Figure S2. Cobimetinib and Trametinib inhibited the oncolytic effect and replication of M1 virus.**


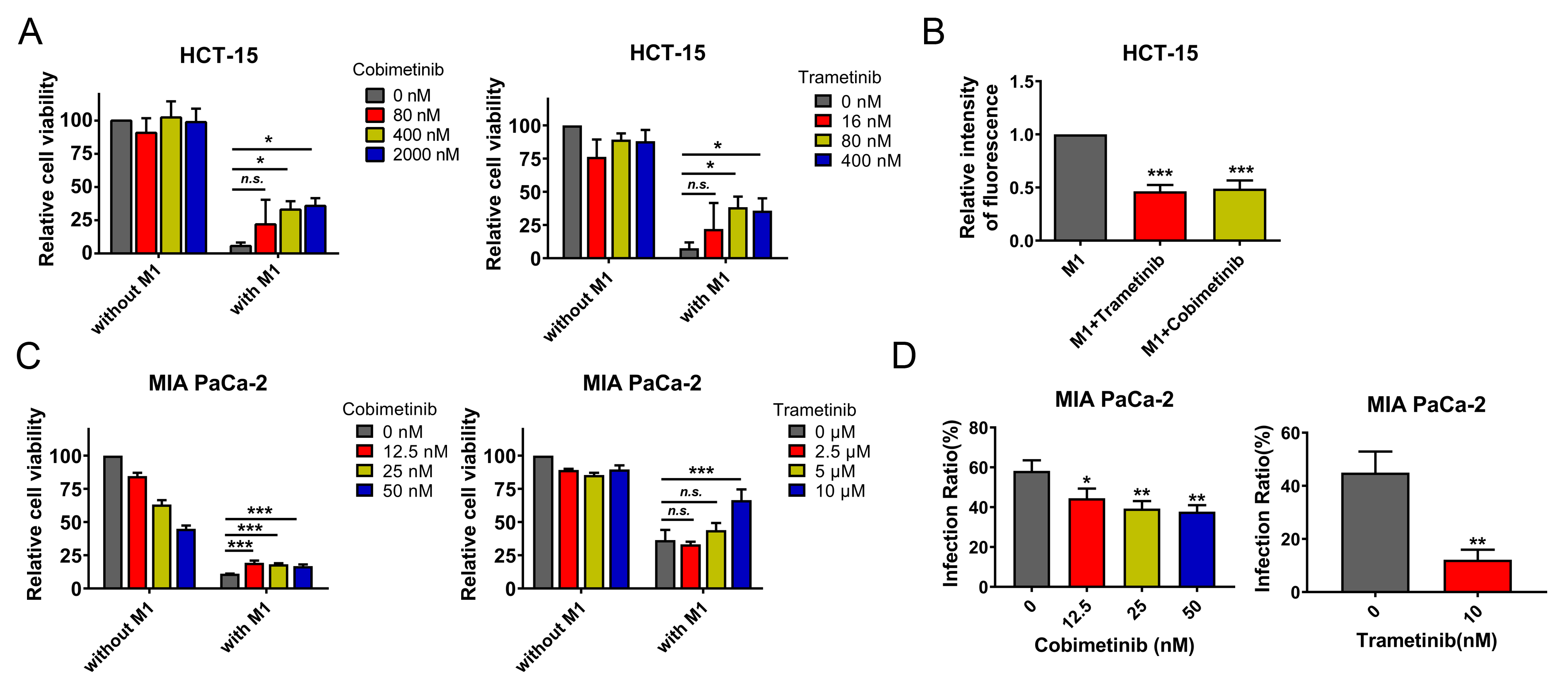


(A) HCT-15 cell line was treated with different concentrations of Cobimetinib or Trametinib with or without M1 (MOI=1 pfu/cell) for 60 hours, cell viability was detected by MTT. n=3. Statistical analysis was performed by one way ANOVA with Dunnett’s tests for pairwise comparisons.

(B) HCT-15 cell line was treated with M1 (MOI=1 pfu/cell), Cobimetinib (400 nM) plus M1 (MOI=1 pfu/cell), or Trametinib plus M1 (MOI=1 pfu/cell) for 24 hours, the intensity of GFP fluorescence was detected. n=3. Statistical analysis was performed by one way ANOVA with Dunnett’s tests for pairwise comparisons.

(C) MIA PaCa-2 cell line was treated with different concentrations of Cobimetinib or Trametinib with or without M1 (MOI=1 pfu/cell) for 60 hours, cell viability was detected by MTT. n=3. Statistical analysis was performed by one way ANOVA with Dunnett’s tests for pairwise comparisons.

(D) MIA PaCa-2 cell line was treated with M1 (MOI=1 pfu/cell), Cobimetinib plus M1 (MOI=1 pfu/cell), or Trametinib (10 μM) plus M1 (MOI=1 pfu/cell) for 24 hours, infection rate of M1 virus (GFP percentage) was detected by flow cytometry. n=3. Statistical analysis was performed by two tailed Student’s *t* test or one way ANOVA with Dunnett’s tests for pairwise comparisons.

Error bars represent mean ± SD obtained from three independent experiments with three technical replications. *n.s.*, not significant; **p*<0.05, ** *p*<0.01, *** *p*<0.001.

**Table S3. Genes regulated by *k-Ras* activation in expression profile database. Refers to Figure 3.**

| Gene Symbol | Gene Symbol | | Gene Symbol | Gene Symbol | Gene Symbol | Gene Symbol |
| --- | --- | --- | --- | --- | --- | --- |
| ANGPTL4 | WNT7A | LY96 | | CFB | CPE | FUCA1 |
| ITGA2 | IGFBP3 | FLT4 | | BTC | SPARCL1 | PLVAP |
| SPRY2 | SPP1 | SPON1 | | PPP1R15A | ABCB1 | ADAM17 |
| HBEGF | ETV1 | BMP2 | | PTPRR | USH1C | AVL9 |
| RBP4 | CLEC4A | PLEK2 | | CCL20 | CSF2RA | ADAMDEC1 |
| HSD11B1 | CCND2 | IGF2 | | ARG1 | BTBD3 | HKDC1 |
| ETV4 | TSPAN7 | NR1H4 | | RETN | IL2RG | MAP7 |
| GLRX | ITGBL1 | SNAP25 | | KLF4 | DNMBP | IL7R |
| DUSP6 | EMP1 | ACE | | MMD | IL10RA | RBM4 |
| SCG5 | CDADC1 | PRRX1 | | PDCD1LG2 | EREG | BPGM |
| ETV5 | KIF5C | C3AR1 | | HIST1H2BB | SLMO2 | ENG |
| ITGB2 | TRIB2 | TRAF1 | | HOXD11 | EPHB2 | GFPT2 |
| AKT2 | SDCCAG8 | TLR8 | | TRIB1 | FBXO4 | PLAU |
| PPBP | PCP4 | ID2 | | F2RL1 | CROT | GNG11 |
| G0S2 | CFHR2 | TMEM100 | | ANXA10 | MPZL2 | PTCD2 |
| GABRA3 | ALDH1A2 | PLAUR | | TSPAN13 | ANKH | MAP3K1 |
| IRF8 | NR0B2 | GADD45G | | MTMR10 | CBR4 | CBL |
| BIRC3 | ALDH1A3 | CBX8 | | CFH | DOCK2 | CXCR4 |
| FGF9 | AMMECR1 | SCN1B | | LAT2 | GPRC5B | NIN |
| DCBLD2 | SATB1 | PTBP2 | | ERO1L | RABGAP1L | IKZF1 |
| INHBA | GUCY1A3 | NAP1L2 | | RELN | MALL | WDR33 |
| TFPI | CSF2 | AKAP12 | | KCNN4 | STRN | MYCN |
| TSPAN1 | APOD | PLAT | | TMEM176A | ST6GAL1 | FCER1G |
| ADAM8 | TOR1AIP2 | SCG3 | | MAP4K1 | PIGR | PECAM1 |
| SLPI | CMKLR1 | ANO1 | | PTGS2 | VWA5A | FAM190B |
| PRKG2 | TMEM176B | IL1RL2 | | IL33 | PSMB8 | SNAP91 |
| MMP11 | GPR124 | CXCL10 | | MAFB | F13A1 | EVI5 |
| MMP10 | LAPTM5 | ATG10 | | LCP1 | NRP1 | TNFRSF1B |
| TMEM158 | CD37 | YRDC | | NGF | SOX9 | GPNMB |
| TNFAIP3 | CAB39L | HDAC9 | | CA2 | JUP | TPH1 |
| PRDM1 | CIDEA | PEG3 | | SERPINA3 | ELTD1 |  |
| GALNT3 | ZNF639 | SEMA3B | | RGS16 | ZNF277 |  |
| ETS1 | IL1B | TNNT2 | | CTSS | EPB41L3 |  |
| MMP9 | GYPC | LIF | | USP12 | PCSK1N |  |

**Table S4. Interferon alpha response genes in expression profile database. Refers to Figure 3.**

| Gene Symbol | | Gene Symbol | | Gene Symbol | |
| --- | --- | --- | --- | --- | --- |
| 1 | MX1 | 34 | CMPK2 | 67 | GBP4 |
| 2 | ISG15 | 35 | IFITM3 | 68 | NCOA7 |
| 3 | OAS1 | 36 | RTP4 | 69 | TMEM140 |
| 4 | IFIT3 | 37 | STAT2 | 70 | CD74 |
| 5 | IFI44 | 38 | SAMD9L | 71 | GMPR |
| 6 | IFI35 | 39 | LY6E | 72 | PSMA3 |
| 7 | IRF7 | 40 | IFITM2 | 73 | PROCR |
| 8 | RSAD2 | 41 | PRIC285 | 74 | IL7 |
| 9 | IFI44L | 42 | CXCL11 | 75 | IFI30 |
| 10 | IFITM1 | 43 | TRIM21 | 76 | IRF2 |
| 11 | IFI27 | 44 | PARP14 | 77 | CSF1 |
| 12 | IRF9 | 45 | TRIM26 | 78 | IL15 |
| 13 | OASL | 46 | PARP12 | 79 | CNP |
| 14 | EIF2AK2 | 47 | NMI | 80 | FAM46A |
| 15 | IFIT2 | 48 | RNF31 | 81 | IL4R |
| 16 | CXCL10 | 49 | HLA-C | 82 | FTSJD2 |
| 17 | TAP1 | 50 | CASP1 | 83 | CD47 |
| 18 | SP110 | 51 | TRIM14 | 84 | LPAR6 |
| 19 | DDX60 | 52 | TDRD7 | 85 | MOV10 |
| 20 | UBE2L6 | 53 | DHX58 | 86 | CASP8 |
| 21 | USP18 | 54 | PARP9 | 87 | TXNIP |
| 22 | PSMB8 | 55 | PNPT1 | 88 | SLC25A28 |
| 23 | IFIH1 | 56 | TRIM25 | 89 | SELL |
| 24 | BST2 | 57 | PSME1 | 90 | TRAFD1 |
| 25 | LGALS3BP | 58 | WARS | 91 | BATF2 |
| 26 | ADAR | 59 | EPSTI1 | 92 | RIPK2 |
| 27 | ISG20 | 60 | UBA7 | 93 | CCRL2 |
| 28 | GBP2 | 61 | PSME2 | 94 | NUB1 |
| 29 | IRF1 | 62 | B2M | 95 | OGFR |
| 30 | PLSCR1 | 63 | TRIM5 | 96 | FAM125A |
| 31 | PSMB9 | 64 | C1S | 97 | ELF1 |
| 32 | HERC6 | 65 | LAP3 |  |  |
| 33 | SAMD9 | 66 | LAMP3 |  |  |

**Table S5. Interferon beta response genes in expression profile database. Refers to Figure 3.**

| Gene Symbol | Gene Symbol | Gene Symbol | Gene Symbol | Gene Symbol |
| --- | --- | --- | --- | --- |
| STAT1 | PLA2G4A | VCAM1 | HLA-DRB1 | CDKN1A |
| ISG15 | TRIM21 | CD274 | GBP6 | IRF4 |
| IFIT1 | USP18 | CIITA | LCP2 | NFKB1 |
| MX1 | PTGS2 | NAMPT | HLA-G | BATF2 |
| IFIT3 | EPSTI1 | SELP | MT2A | HLA-B |
| IFI35 | C1S | GPR18 | RIPK1 | LATS2 |
| IRF7 | DDX58 | FPR1 | KLRK1 | IRF5 |
| IFIT2 | IL15 | PRIC285 | UPP1 | SLAMF7 |
| OAS2 | NLRC5 | PSME2 | PSMB2 | ISOC1 |
| TAP1 | NMI | SERPING1 | TDRD7 | P2RY14 |
| EIF2AK2 | IDO1 | CCL5 | HIF1A | STAT3 |
| RSAD2 | PSMB10 | RNF31 | EIF4E3 | NCOA3 |
| MX2 | CXCL11 | SOD2 | VAMP8 | HLA-A |
| IRF1 | ITGB7 | TRIM25 | PFKP | IL6 |
| OAS3 | SAMHD1 | LAP3 | CD38 | GZMA |
| TNFSF10 | HERC6 | PSMA3 | ZBP1 | IFNAR2 |
| IRF9 | CMPK2 | RNF213 | BANK1 | CD74 |
| CXCL10 | SAMD9L | PELI1 | TOR1B | RAPGEF6 |
| IFI44 | RTP4 | CFB | RBCK1 | CASP4 |
| BST2 | PTPN2 | CD86 | PDE4B | FAS |
| XAF1 | PARP14 | TXNIP | MVP | OGFR |
| SP110 | TNFAIP2 | HLA-DQA1 | IL7 | ARL4A |
| OASL | IFITM2 | GCH1 | BPGM | SRI |
| PSMB8 | PLSCR1 | PNP | FTSJD2 | LYSMD2 |
| IFI44L | SOCS1 | CCL7 | AUTS2 | CSF2RB |
| IFITM3 | CASP1 | PTPN6 | B2M | ST3GAL5 |
| DDX60 | ICAM1 | SPPL2A | RIPK2 | C1R |
| LGALS3BP | WARS | IL4R | CD69 | CASP3 |
| GBP4 | PSME1 | PNPT1 | MYD88 | CMKLR1 |
| IRF8 | ISG20 | DHX58 | PSMA2 | NFKBIA |
| PSMB9 | IRF2 | BTG1 | PIM1 | METTL7B |
| PML | TRIM14 | CASP8 | NOD1 | ST8SIA4 |
| IFIH1 | FCGR1A | IFI30 | CFH | XCL1 |
| UBE2L6 | 1-Mar | CCL2 | TAPBP | IL2RB |
| IFI27 | SOCS3 | FGL2 | SLC25A28 | VAMP5 |
| ADAR | JAK2 | CASP7 | PTPN1 | IL18BP |
| LY6E | HLA-DMA | SECTM1 | TNFAIP3 | ZNFX1 |
| STAT2 | PARP12 | IL15RA | SSPN | ARID5B |
| CXCL9 | TNFAIP6 | CD40 | NUP93 | APOL6 |
| IL10RA | TRIM26 | TRAFD1 | MTHFD2 | STAT4 |

**Figure S3. The efficiency of siRNAs. Refers to Figure 4.**


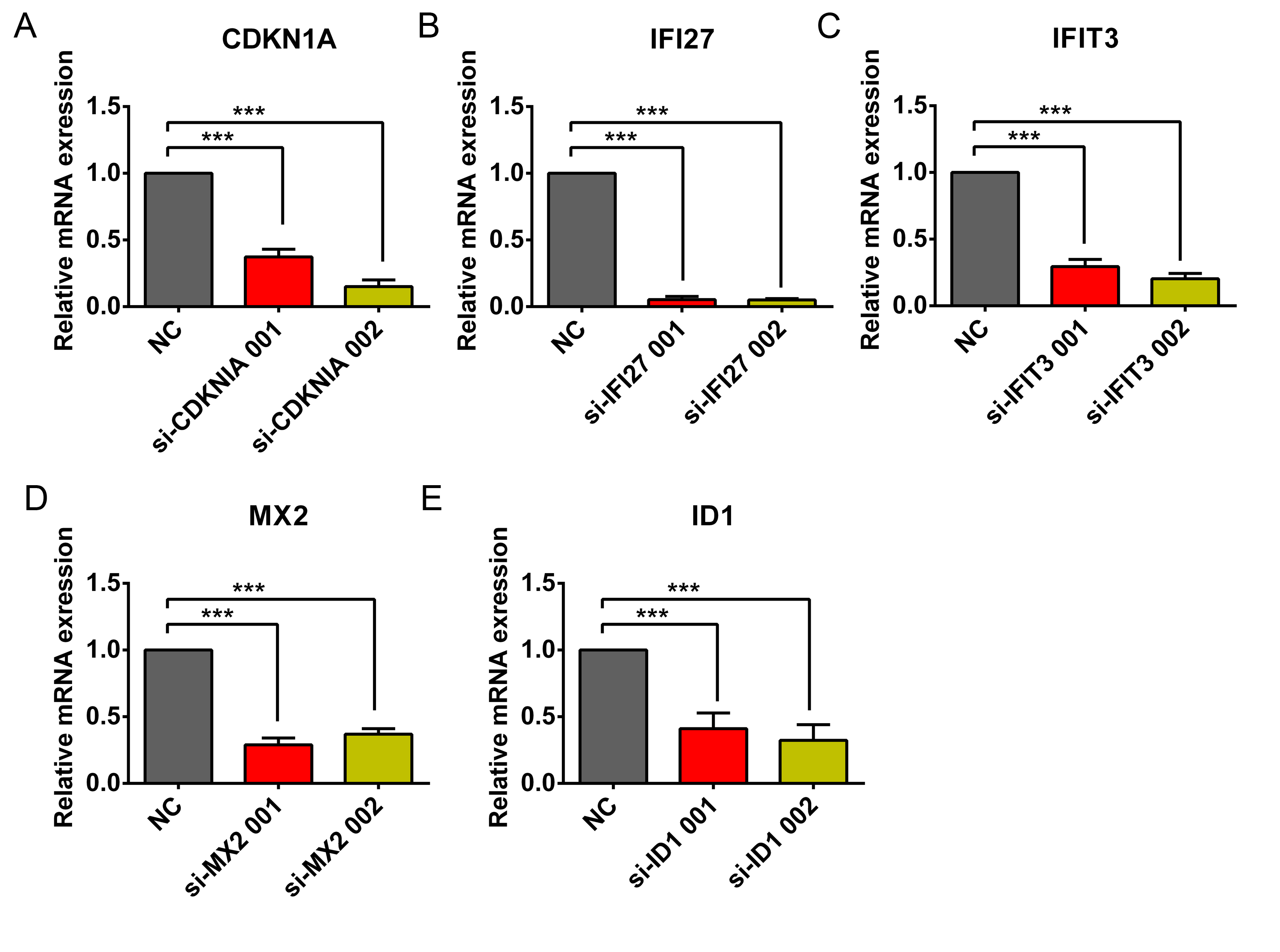


The efficiency of siRNAs to CDKN1A (A), IFI27 (B), IFIT3 (C), MX2 (D) and ID1 (E) in HCT-15 cell line. HCT-15 cells were treated with siRNAs to these genes for 48 hours, relative expression of these gene was detected by RT-qPCR. Error bars represent mean ± SD obtained from three independent experiments with three biological replications. Statistical analysis was performed by one way ANOVA with Dunnett’s tests for pairwise comparisons. **p*<0.05, ** *p*<0.01, *** *p*<0.001.

**Figure S4. Protein level of CDKN1A. Refers to Figure 7.**


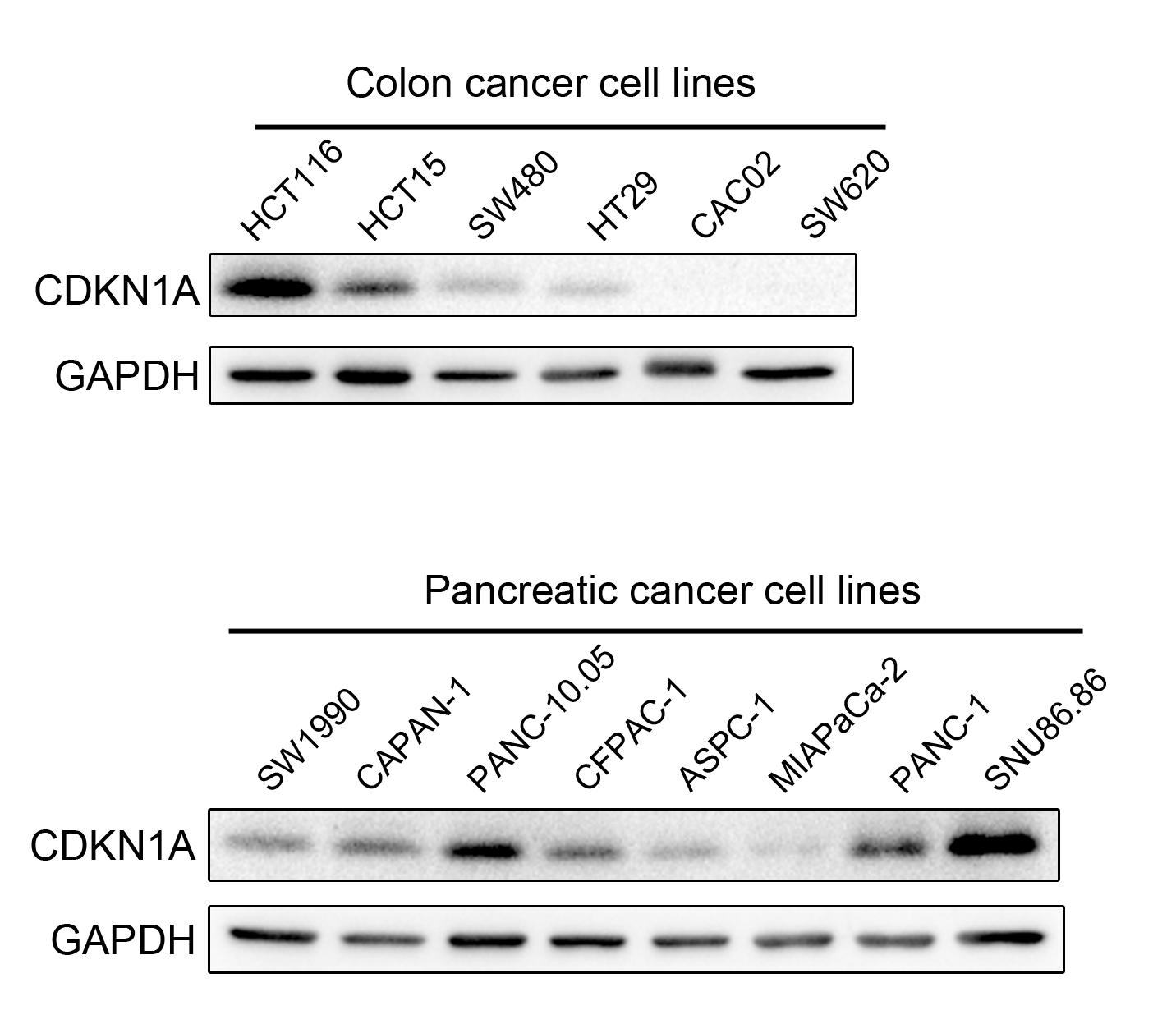


Expression of CDKN1A in colon cancer cell lines and pancreatic cancer cell lines was detected by western blot.

**Table S6. Oncolytic effect of M1 virus in colon cancer cell lines and pancreatic cancer cell lines. Refers to Figure 7.**

| Colon cancer cells | IC50 | Pancreatic cancer cells | Oncolytic effect |
| --- | --- | --- | --- |
| HCT116 | 10.49 | SW1990 | 47.77 |
| HCT15 | 2.009 | CAPAN-1 | 33.64 |
| SW480 | 1.043 | PANC10.05 | 20.32 |
| HT29 | 3 | CFPAC-1 | 32.82 |
| CAC02-2 | 0.005 | ASPC-1 | 56.54 |
| SW620 | 0.015 | MIAPACA-2 | 45.99 |
|  |  | PANC-1 | 35.56 |
|  |  | SNU86.86 | 32.93 |

Colon cancer cell lines were treated with different MOI (MOI= 10, 1, 0.1, 0.01, 0.001,0 pfu/cell) of M1 virus for 72 hours, cell viability was detected by MTT. The viability-dose curve was fitted by Graphpad, and IC50 (half maximal inhibitory concentration) was calculated. n=3. Pancreatic cancer cell lines were treated with (MOI=1pfu/cell) of M1 virus for 72 hours, the oncolytic effect of M1 was indicated by the cell killing percentage detected by MTT. n=3. The results were from three independent experiments with three technical replications.

**Table S7. Oncolytic effect of M1 virus and the protein expression of CDKN1A in 44 tumor cells. Refers to Figure 7.**

Table S7 shows the oncolytic effect of M1 virus and the protein expression of CDKN1A measured by Reverse Phase Protein Arrays (RPPA) in the CCLE data in 44 tumor cells. The oncolytic effect of M1 was indicated by the cell killing percentage detected by MTT from the 44 tumor cell lines treated with M1 virus (MOI=10 pfu/cell) for 48 hours. The data was shown as an additional excel file with the manuscript.
